# Supplementary figures and images for: microRNA-mediated regulation of microRNA machinery controls cell fate decisions
Source: eLife. 2021 Oct 1;10:e72289. doi: 10.7554/eLife.72289 (PMC8504967; doi:10.7554/eLife.72289)

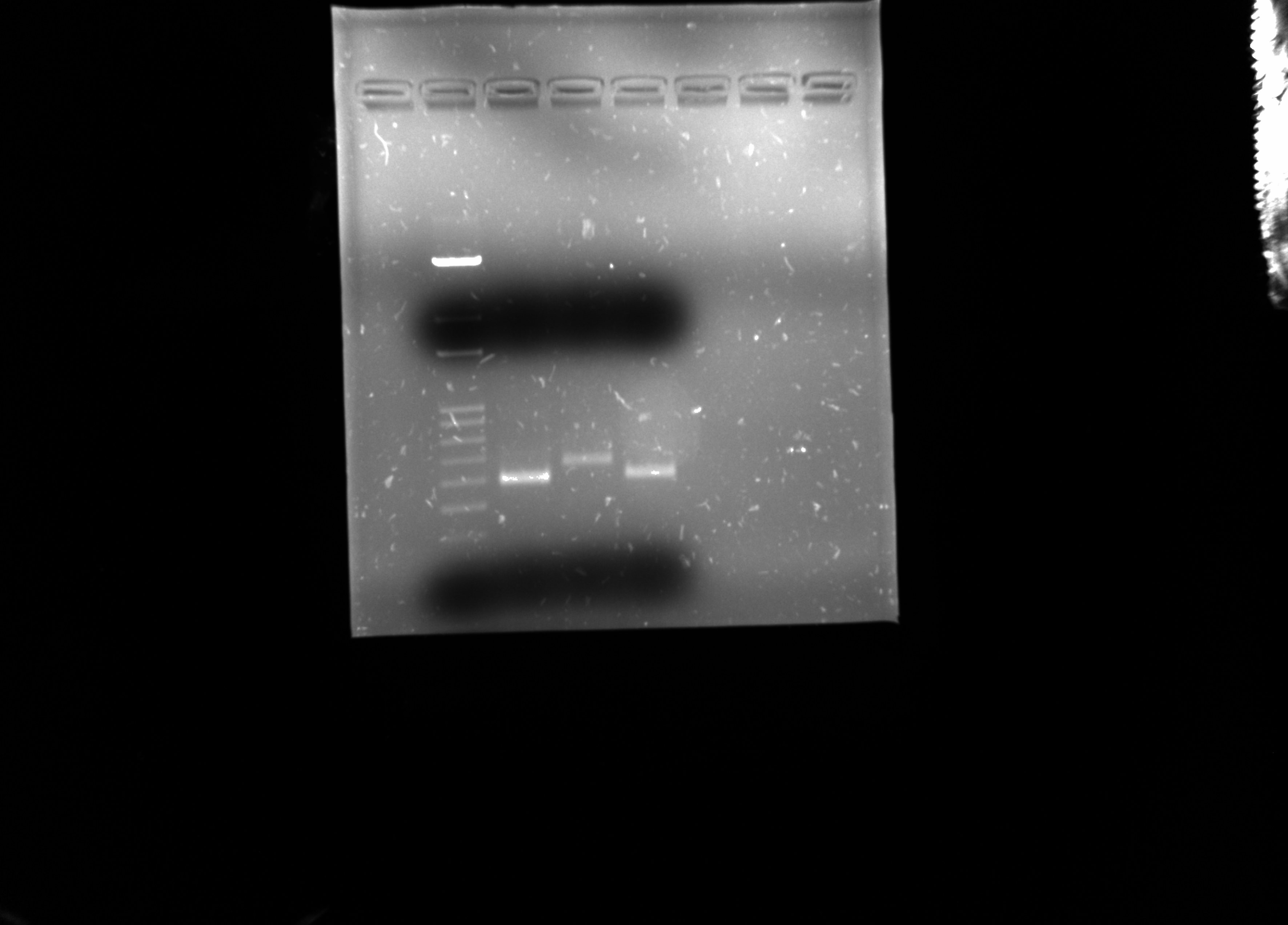

Supplement: Figure 1—source data 1. — Excel files of numbers for Figure 1B and E; Figure 1—figure supplement 1A, D, Figure 1—figure supplement 2. [file elife-72289-fig1-data1.zip › Figure1/Fig1_Fig_Supplement1C_source_data_Ago1_locus.tif]

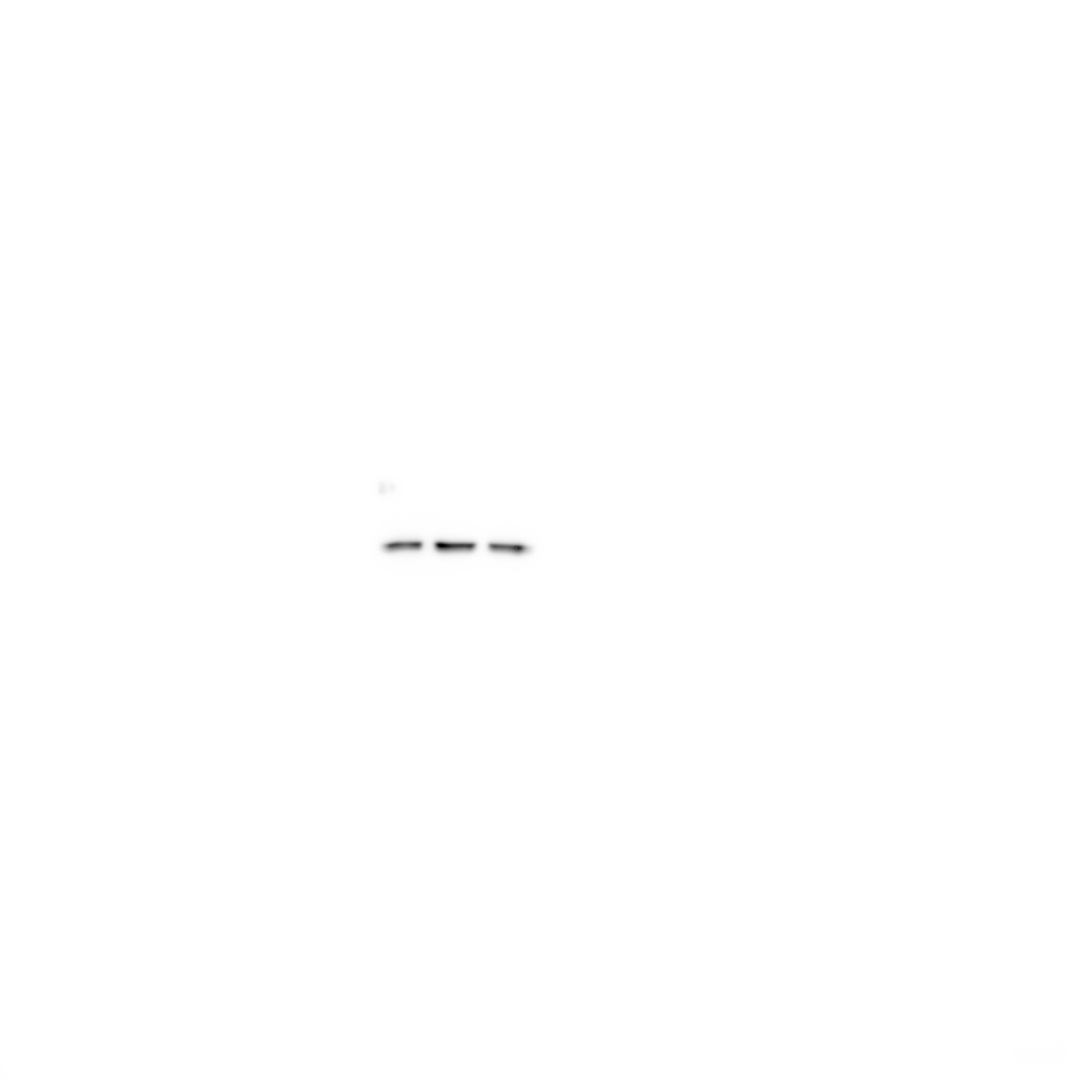

Supplement: Figure 1—source data 1. — Excel files of numbers for Figure 1B and E; Figure 1—figure supplement 1A, D, Figure 1—figure supplement 2. [file elife-72289-fig1-data1.zip › Figure1/Fig1A_source_data_Oct4.tif]

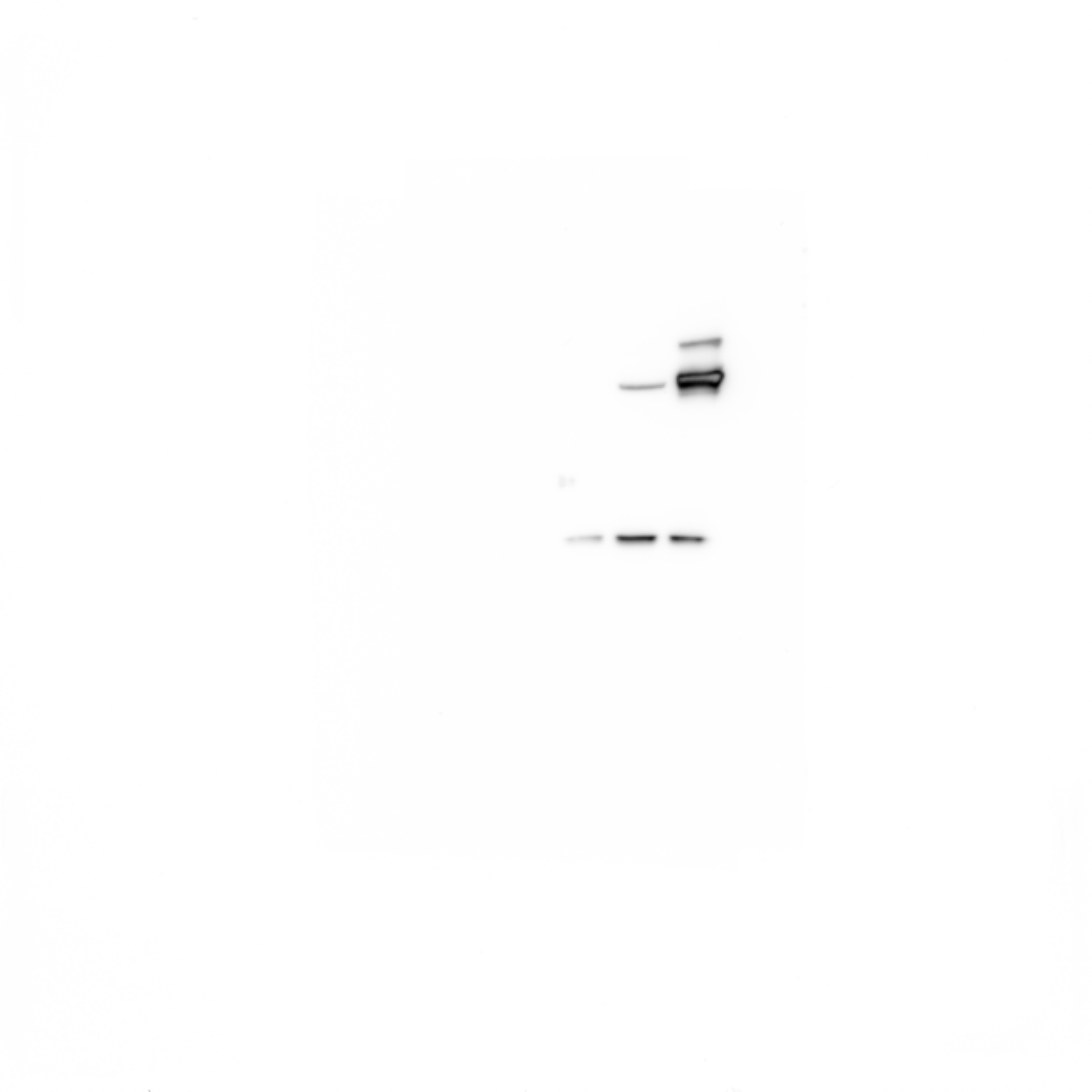

Supplement: Figure 1—source data 1. — Excel files of numbers for Figure 1B and E; Figure 1—figure supplement 1A, D, Figure 1—figure supplement 2. [file elife-72289-fig1-data1.zip › Figure1/Fig1A_source_data_Flag_long.tif]

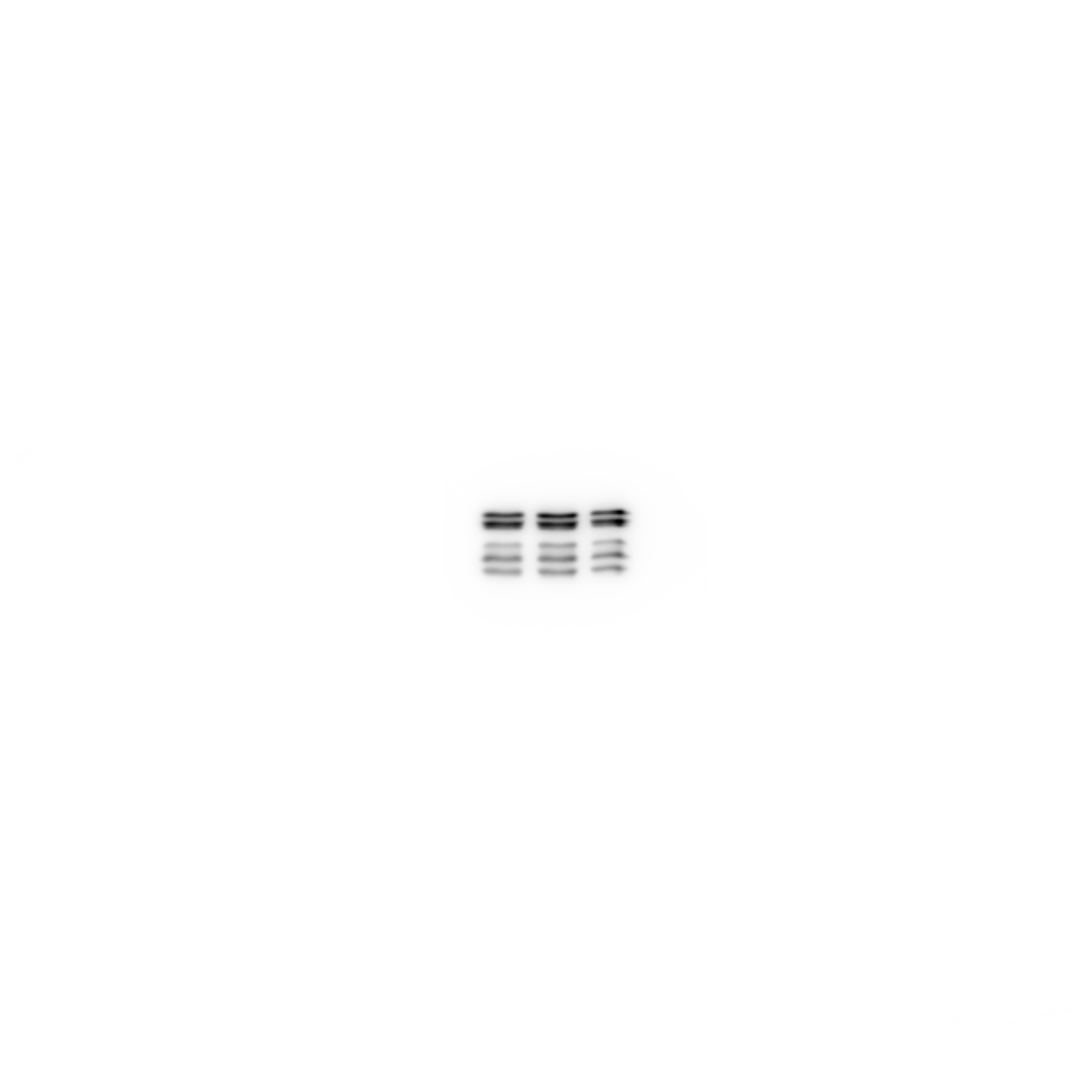

Supplement: Figure 1—source data 1. — Excel files of numbers for Figure 1B and E; Figure 1—figure supplement 1A, D, Figure 1—figure supplement 2. [file elife-72289-fig1-data1.zip › Figure1/Fig1A_source_data_Nanog.tif]

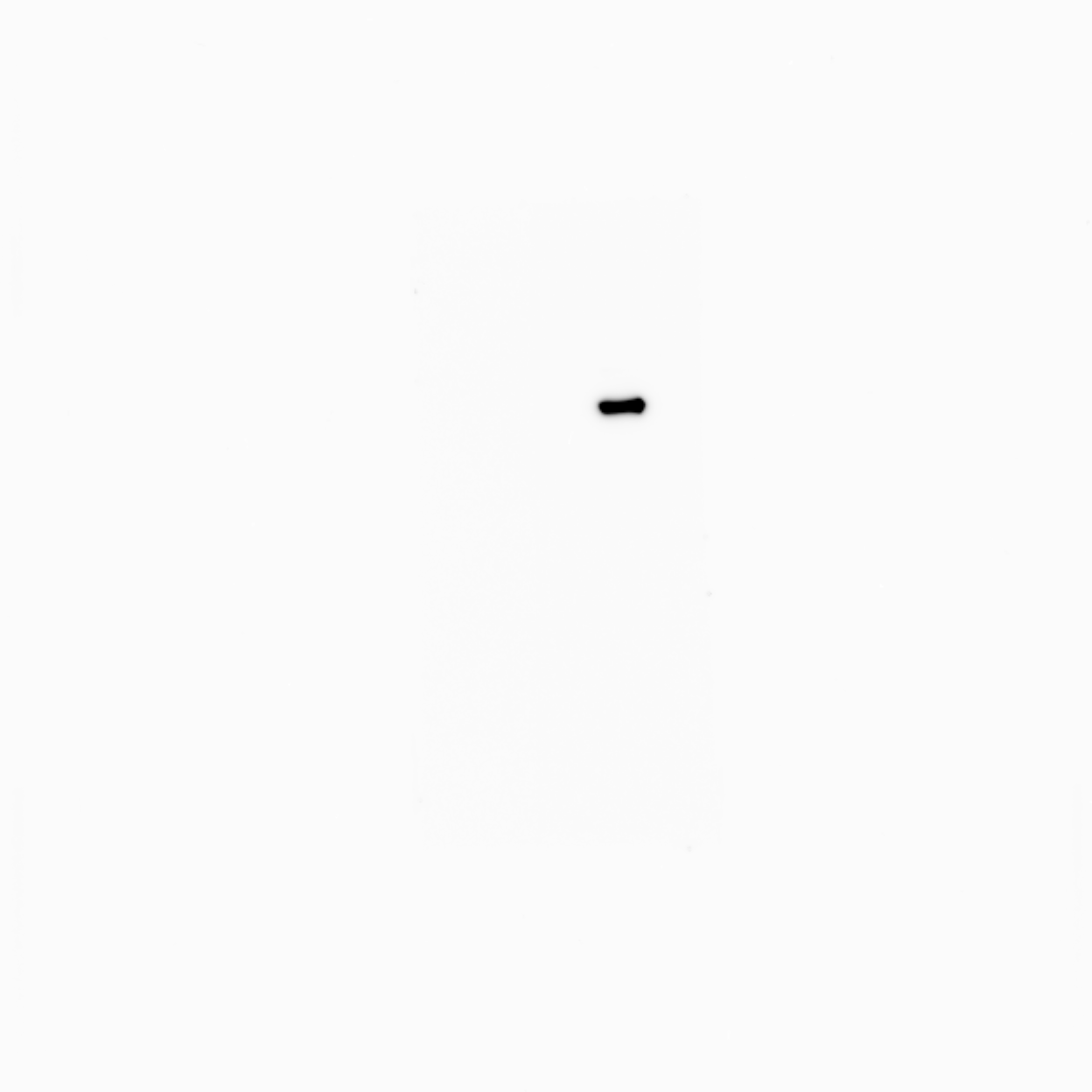

Supplement: Figure 1—source data 1. — Excel files of numbers for Figure 1B and E; Figure 1—figure supplement 1A, D, Figure 1—figure supplement 2. [file elife-72289-fig1-data1.zip › Figure1/Fig1A_source_data_Flag.tif]

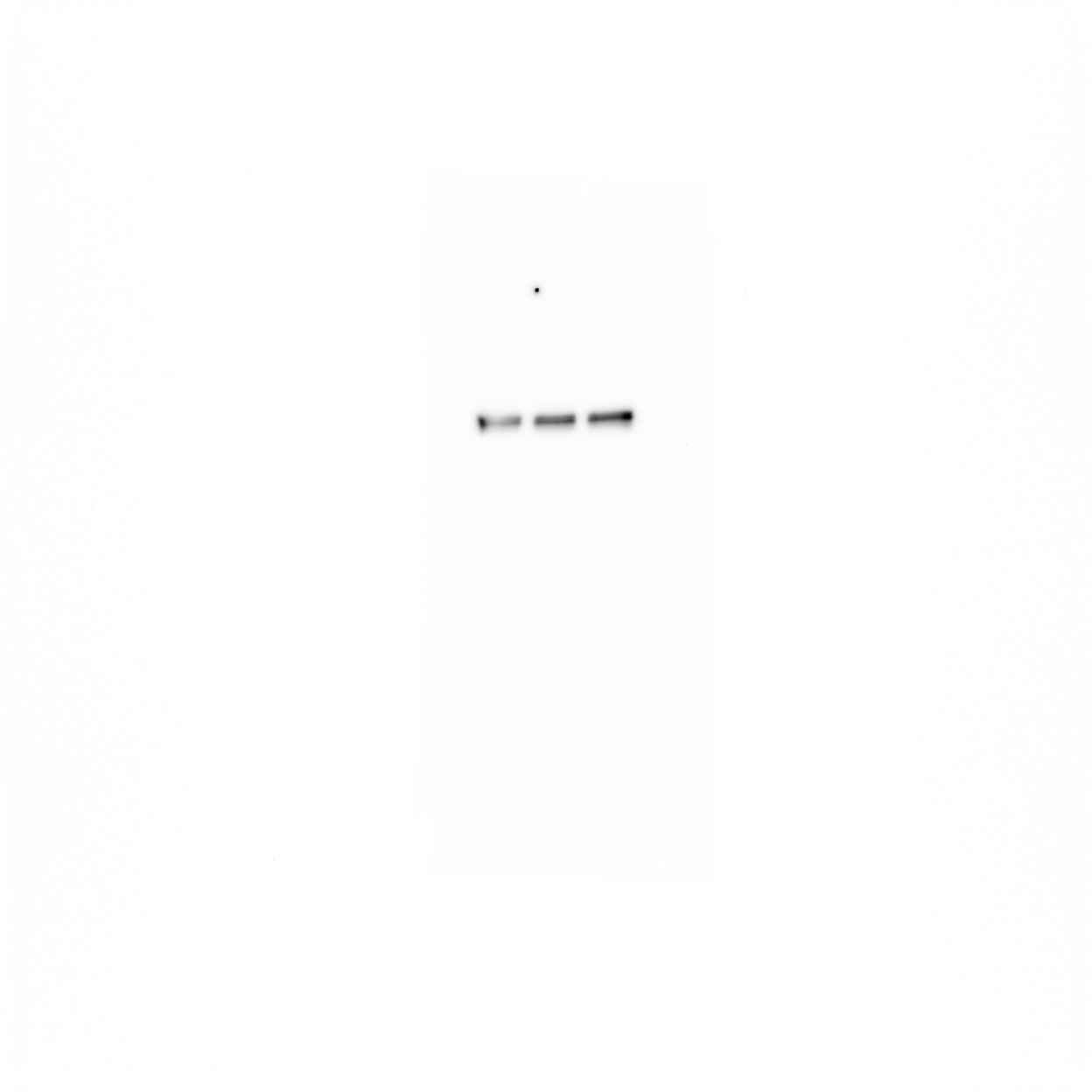

Supplement: Figure 1—source data 1. — Excel files of numbers for Figure 1B and E; Figure 1—figure supplement 1A, D, Figure 1—figure supplement 2. [file elife-72289-fig1-data1.zip › Figure1/Fig1C_source_data_Ago2.tif]

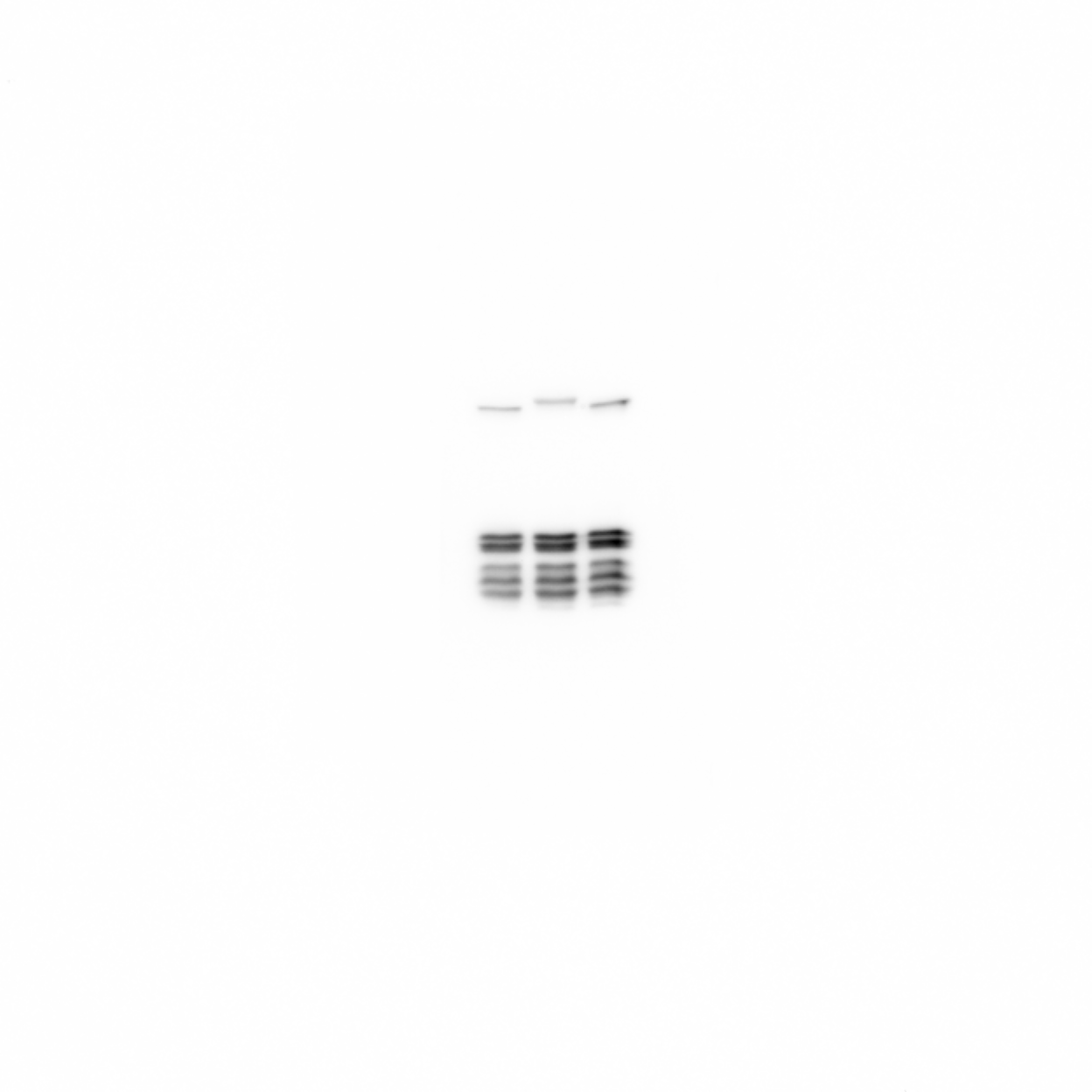

Supplement: Figure 1—source data 1. — Excel files of numbers for Figure 1B and E; Figure 1—figure supplement 1A, D, Figure 1—figure supplement 2. [file elife-72289-fig1-data1.zip › Figure1/Fig1A_source_data_Ago1.tif]

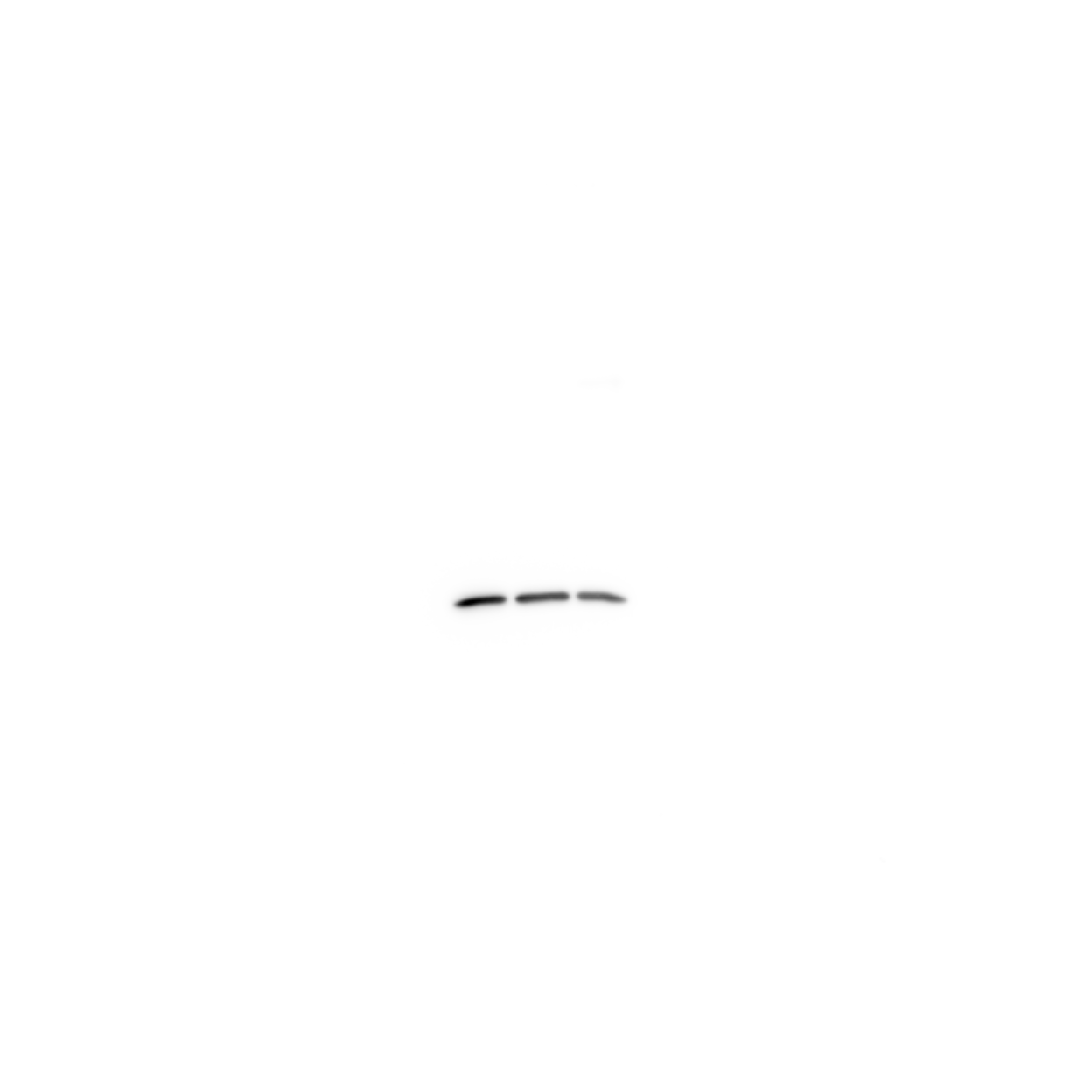

Supplement: Figure 1—source data 1. — Excel files of numbers for Figure 1B and E; Figure 1—figure supplement 1A, D, Figure 1—figure supplement 2. [file elife-72289-fig1-data1.zip › Figure1/Fig1A_source_data_Gapdh.tif]

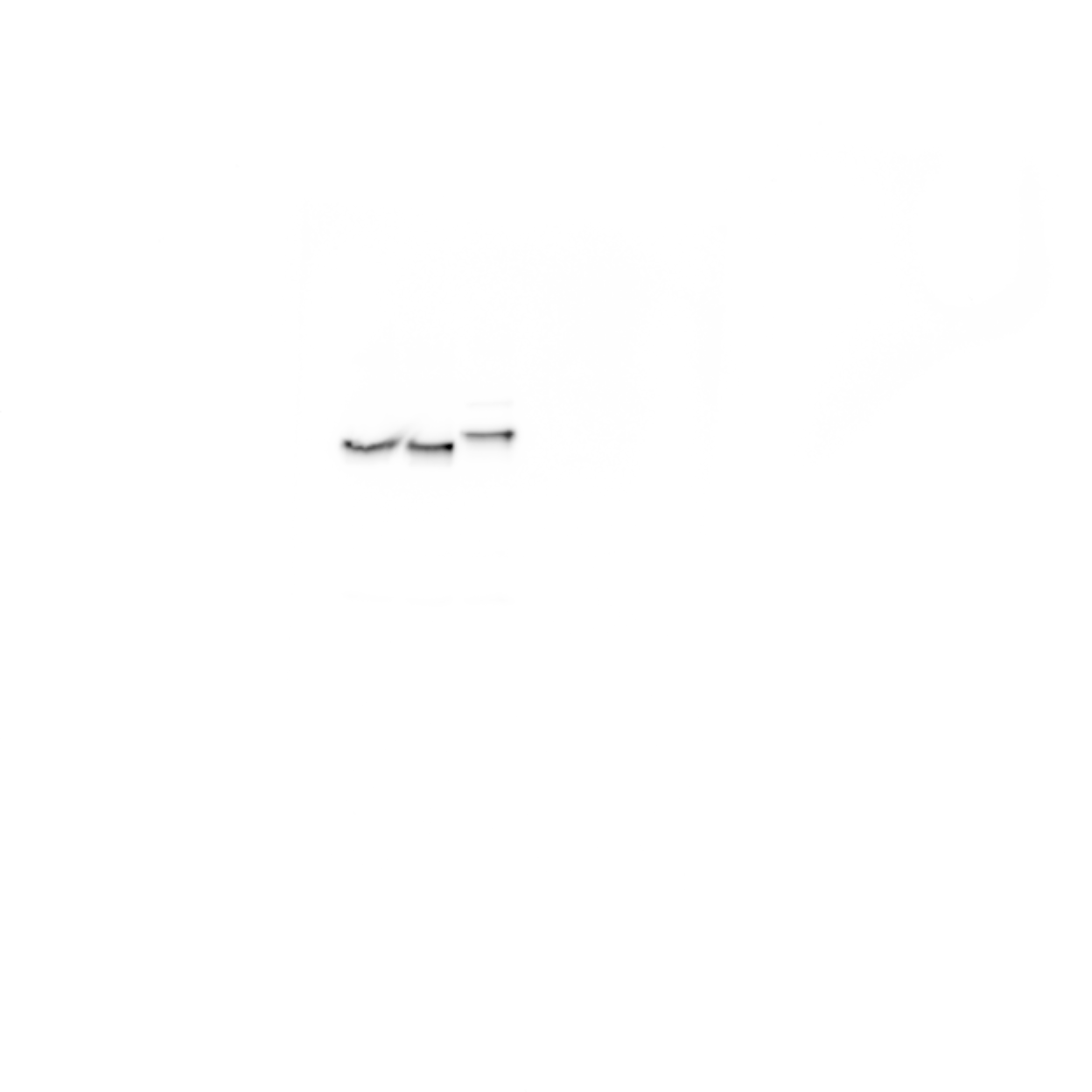

Supplement: Figure 1—source data 1. — Excel files of numbers for Figure 1B and E; Figure 1—figure supplement 1A, D, Figure 1—figure supplement 2. [file elife-72289-fig1-data1.zip › Figure1/Fig1A_source_data_Ago2.tif]

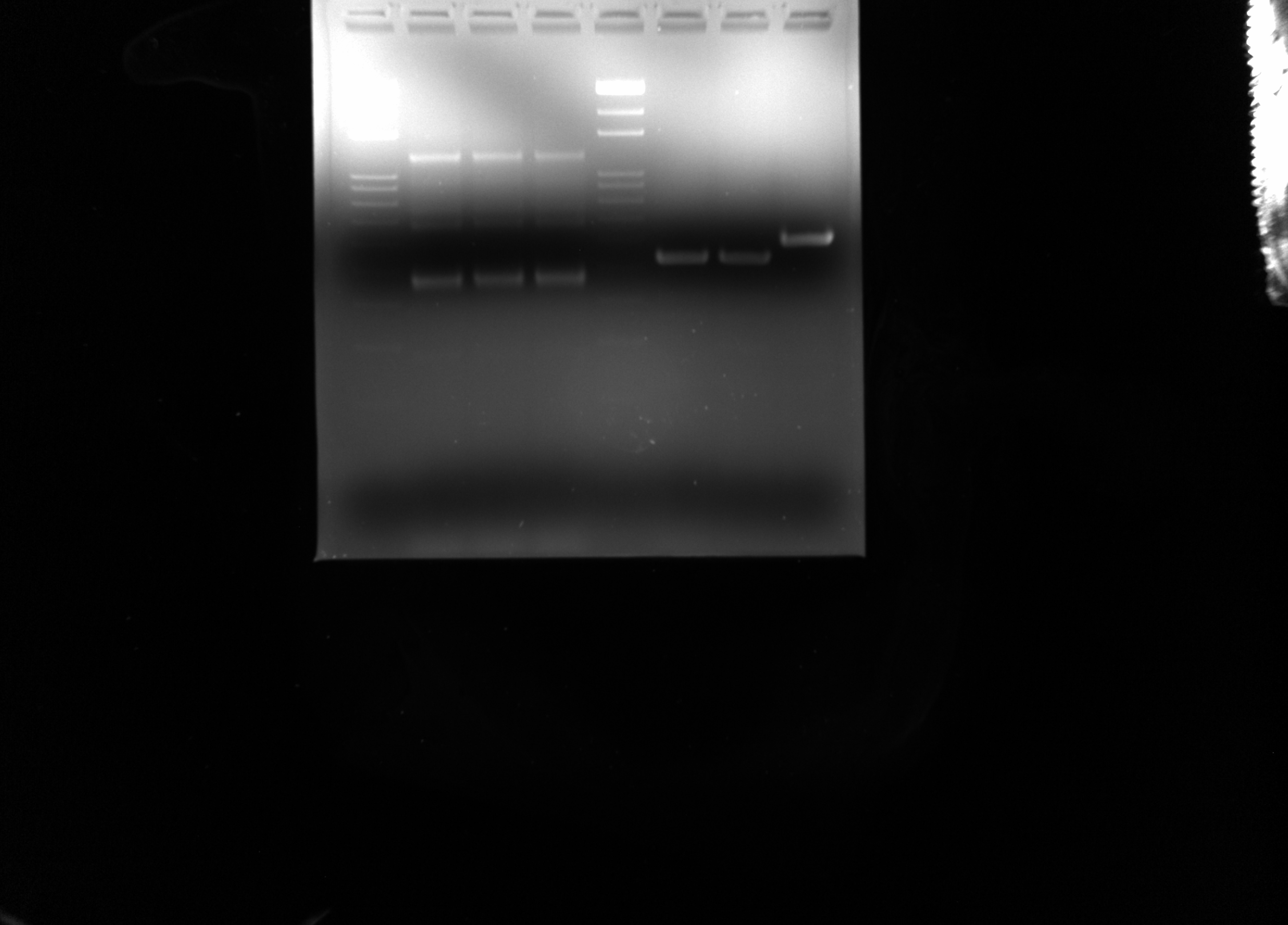

Supplement: Figure 1—source data 1. — Excel files of numbers for Figure 1B and E; Figure 1—figure supplement 1A, D, Figure 1—figure supplement 2. [file elife-72289-fig1-data1.zip › Figure1/Fig1_Fig_Supplement1C_source_data_Ago2_locus.tif]

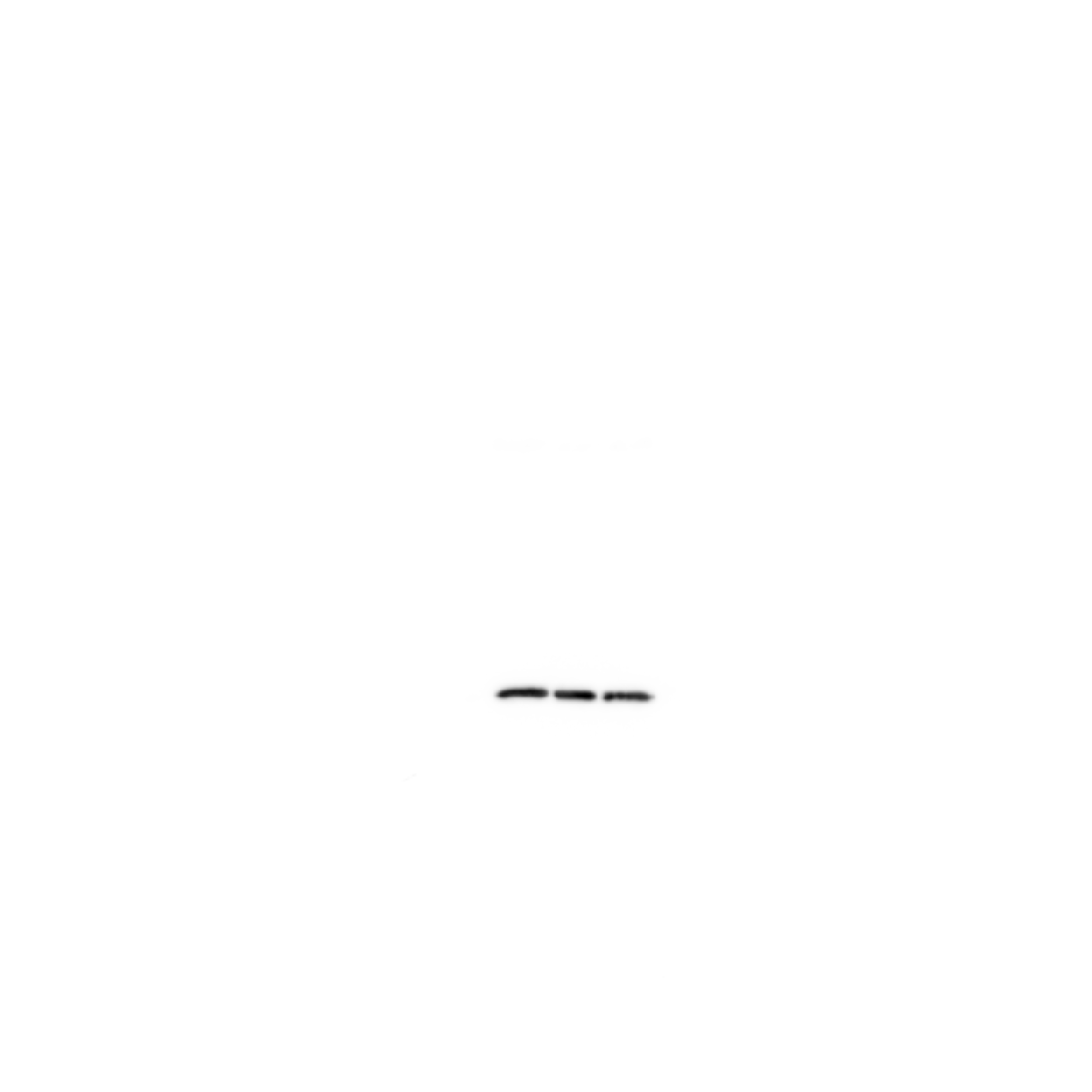

Supplement: Figure 1—source data 1. — Excel files of numbers for Figure 1B and E; Figure 1—figure supplement 1A, D, Figure 1—figure supplement 2. [file elife-72289-fig1-data1.zip › Figure1/Fig1C_source_data_Gapdh.tif]

Ago2

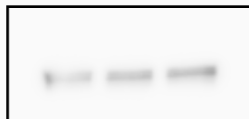

Gapdh

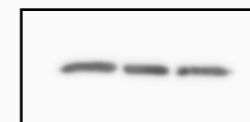

Supplement: Figure 1—source data 1. — Excel files of numbers for Figure 1B and E; Figure 1—figure supplement 1A, D, Figure 1—figure supplement 2. [file elife-72289-fig1-data1.zip › Figure1/Figures with uncropped blots/Figure1C_uncropped.pdf]

Ago1 locus

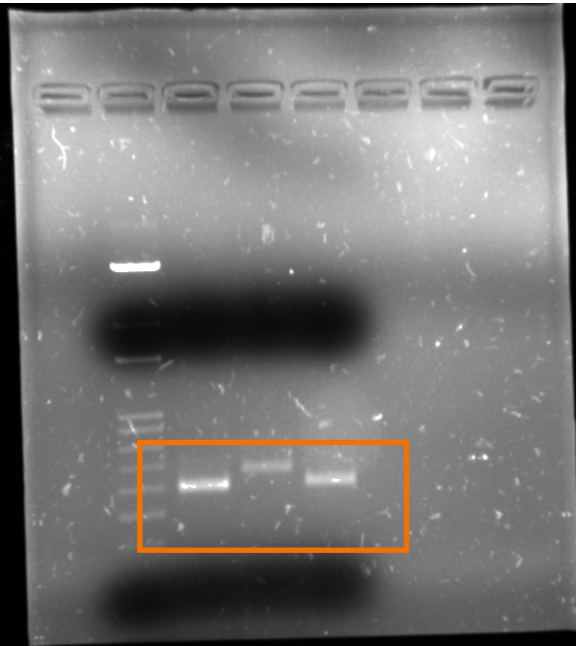

Ago2 locus

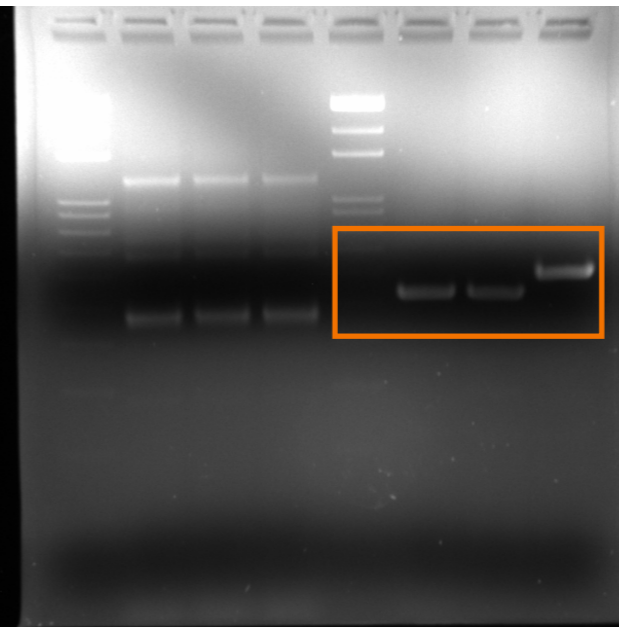

Supplement: Figure 1—source data 1. — Excel files of numbers for Figure 1B and E; Figure 1—figure supplement 1A, D, Figure 1—figure supplement 2. [file elife-72289-fig1-data1.zip › Figure1/Figures with uncropped blots/Figure1_Figure_supplement1C_uncropped.pdf]

anti-FLAG

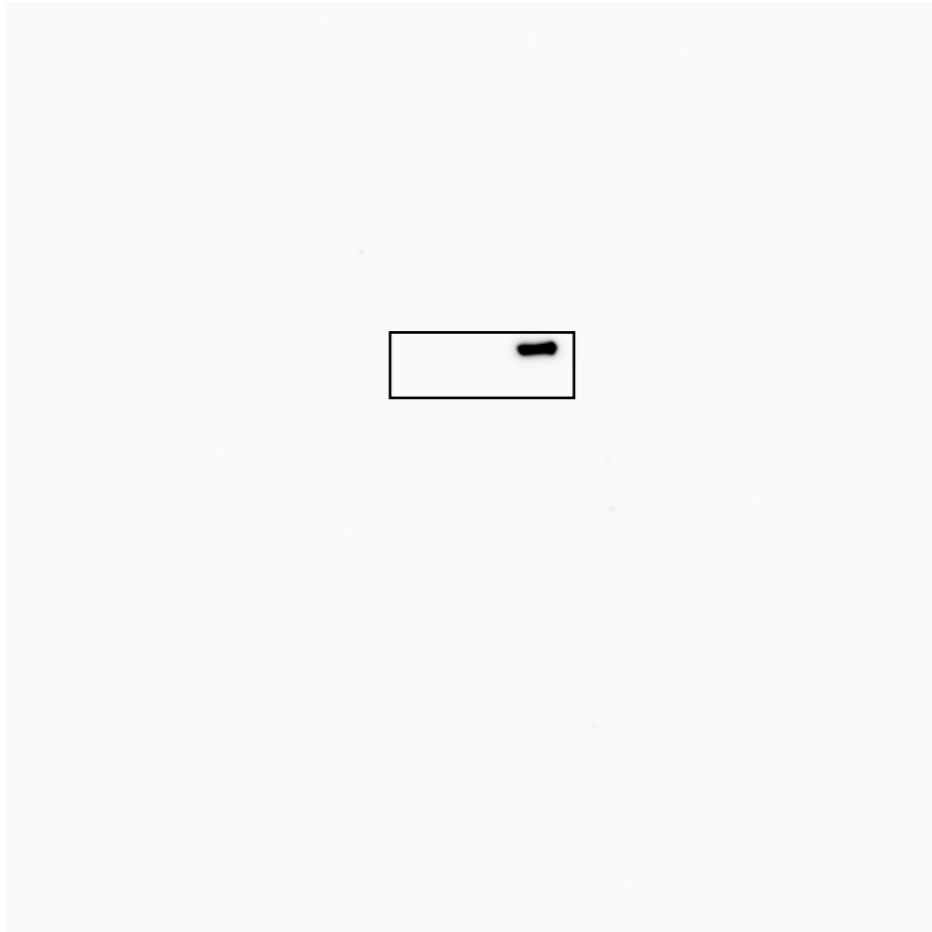

anti-FLAG (long exposure)

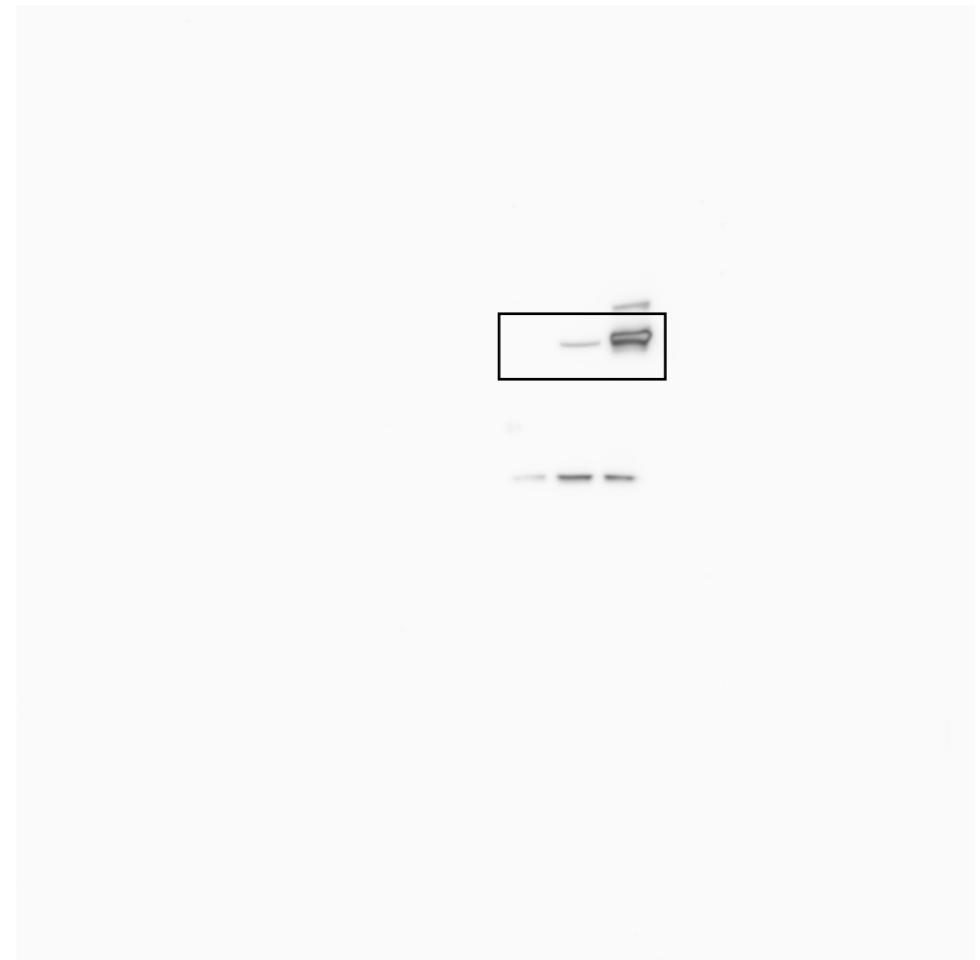

Ago1

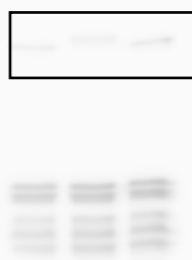

Ago2

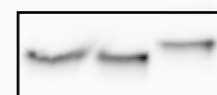

Nanog

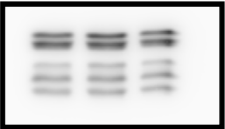

Oct4

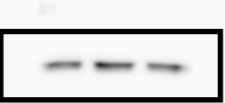

Gapdh

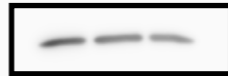

Supplement: Figure 1—source data 1. — Excel files of numbers for Figure 1B and E; Figure 1—figure supplement 1A, D, Figure 1—figure supplement 2. [file elife-72289-fig1-data1.zip › Figure1/Figures with uncropped blots/Figure1A_uncropped.pdf]

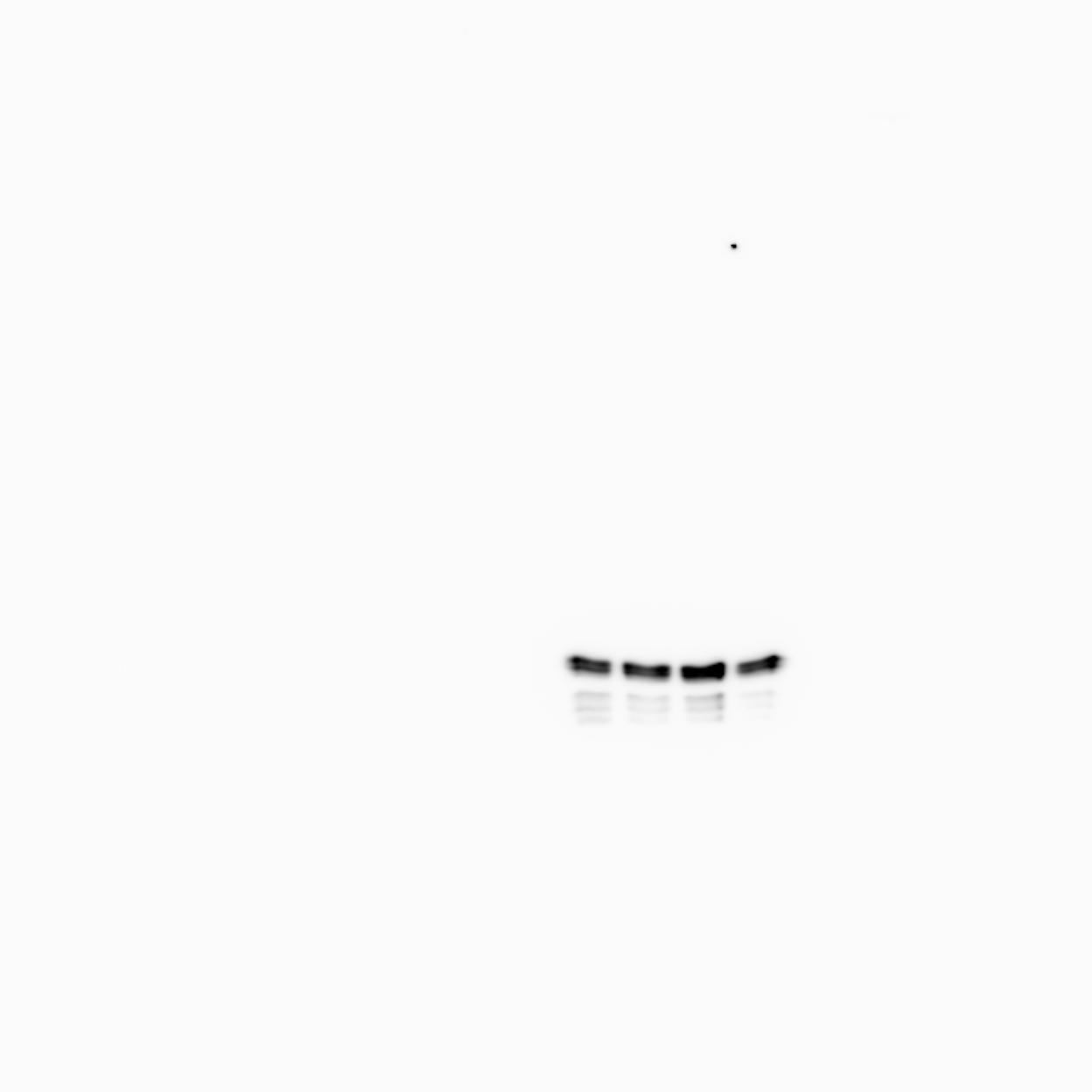

Supplement: Figure 2—source data 1. — Excel files of numbers for Figure 2A, C and D; Figure 2—figure supplement 1B. [file elife-72289-fig2-data1.zip › Figure2/Fig2B__source_data_Nanog.tif]

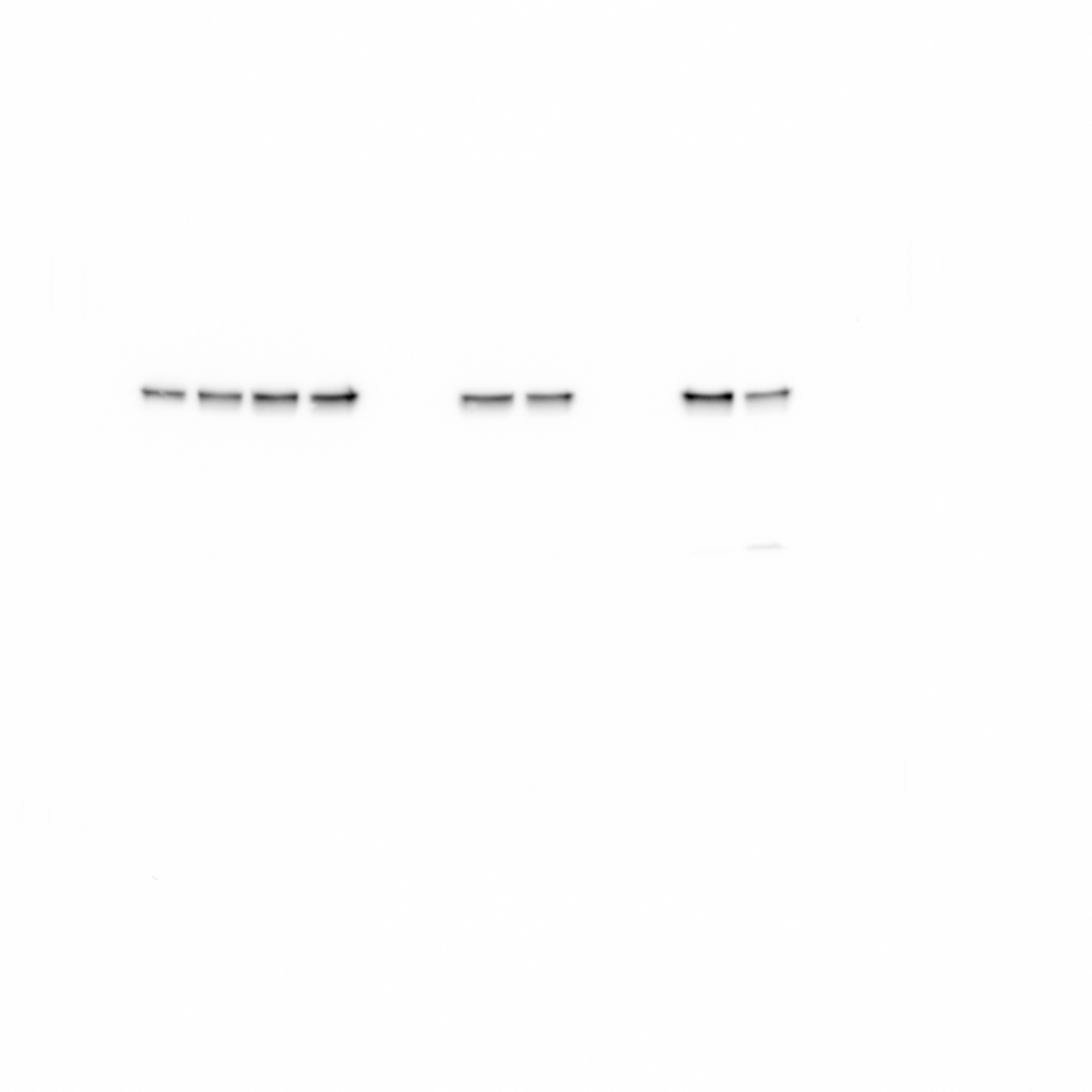

Supplement: Figure 2—source data 1. — Excel files of numbers for Figure 2A, C and D; Figure 2—figure supplement 1B. [file elife-72289-fig2-data1.zip › Figure2/Fig2B__source_data_Ago2.tif]

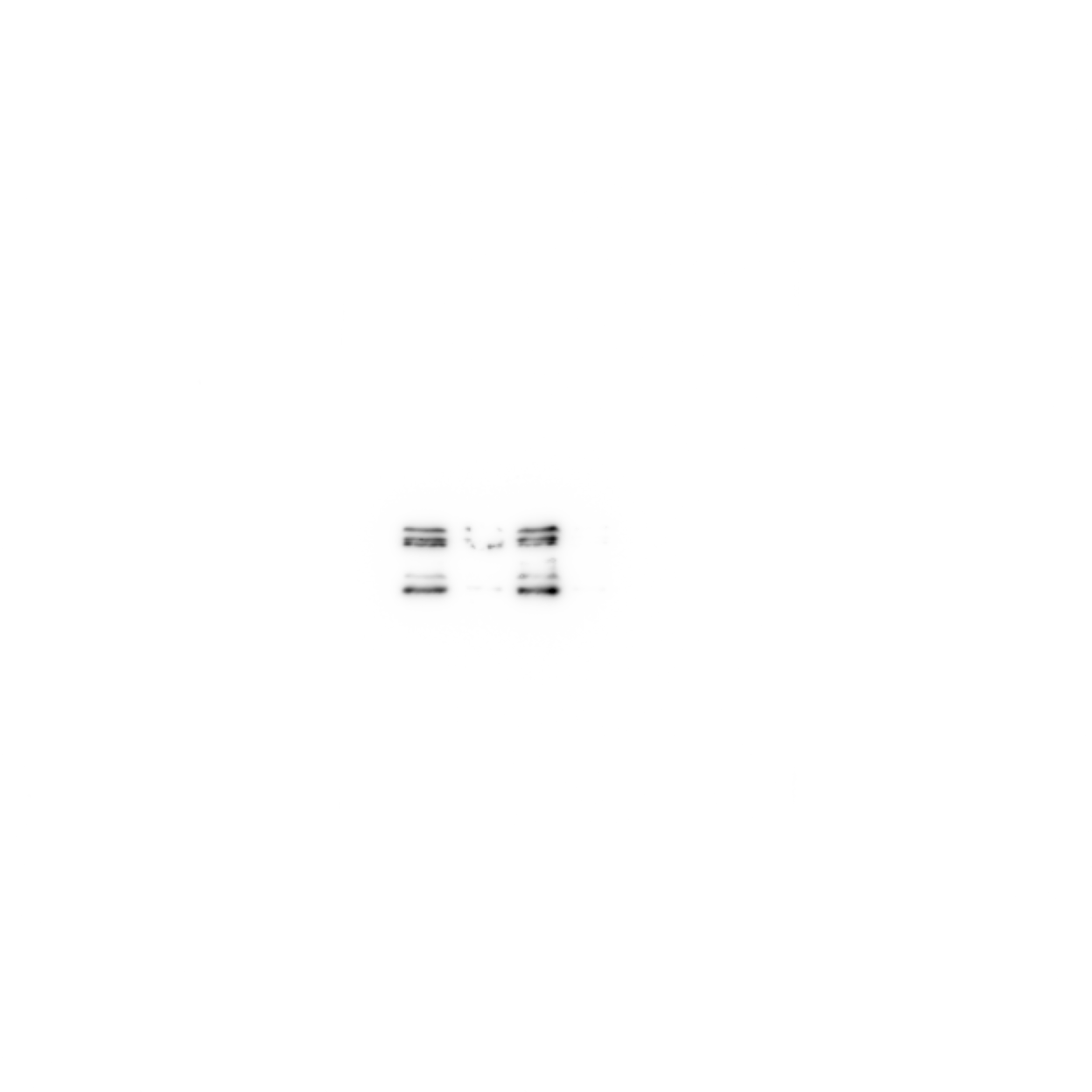

Supplement: Figure 2—source data 1. — Excel files of numbers for Figure 2A, C and D; Figure 2—figure supplement 1B. [file elife-72289-fig2-data1.zip › Figure2/Fig2E_source_data_Nanog.tif]

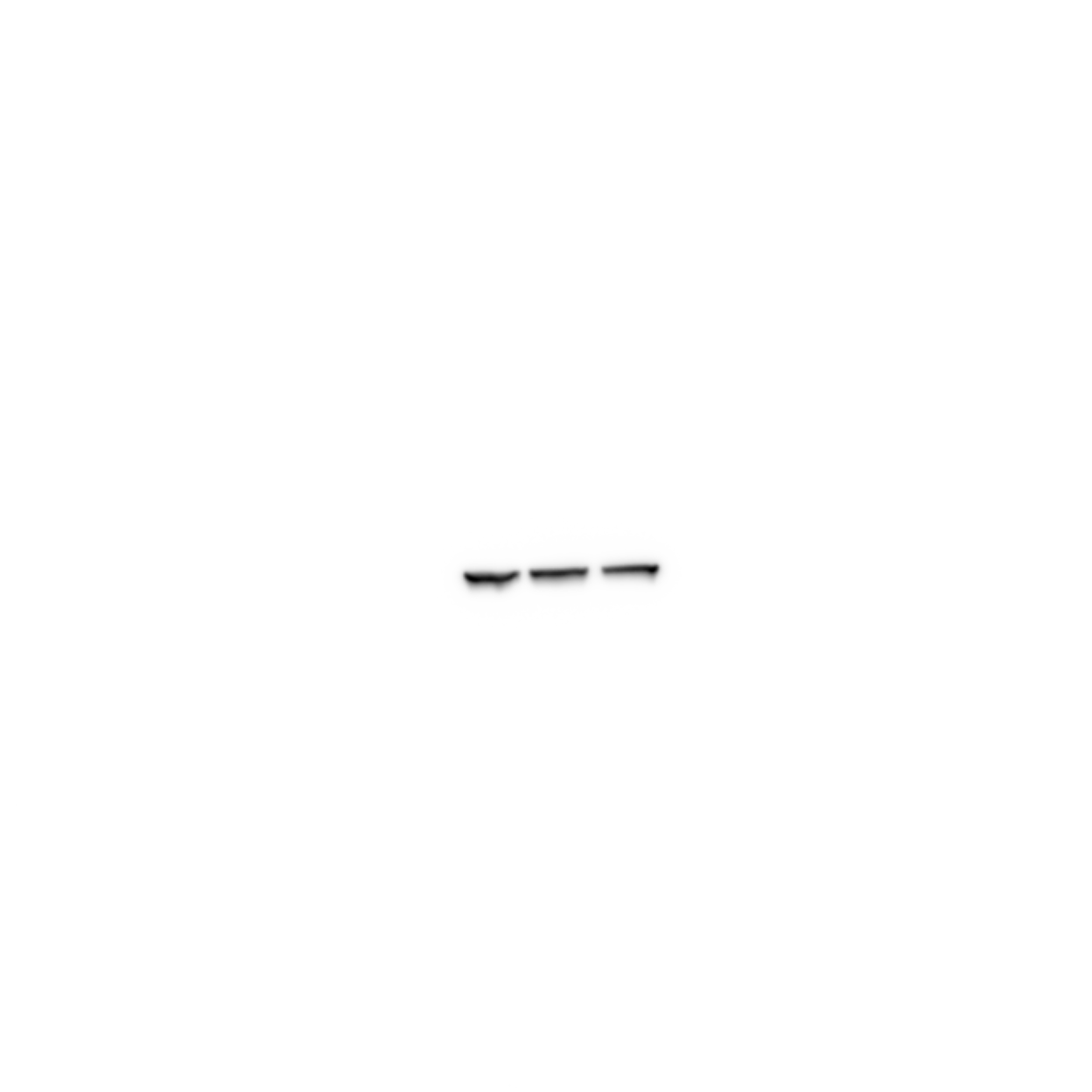

Supplement: Figure 2—source data 1. — Excel files of numbers for Figure 2A, C and D; Figure 2—figure supplement 1B. [file elife-72289-fig2-data1.zip › Figure2/Fig2_Fig_Supplement1C_source_data_Gapdh.tif]

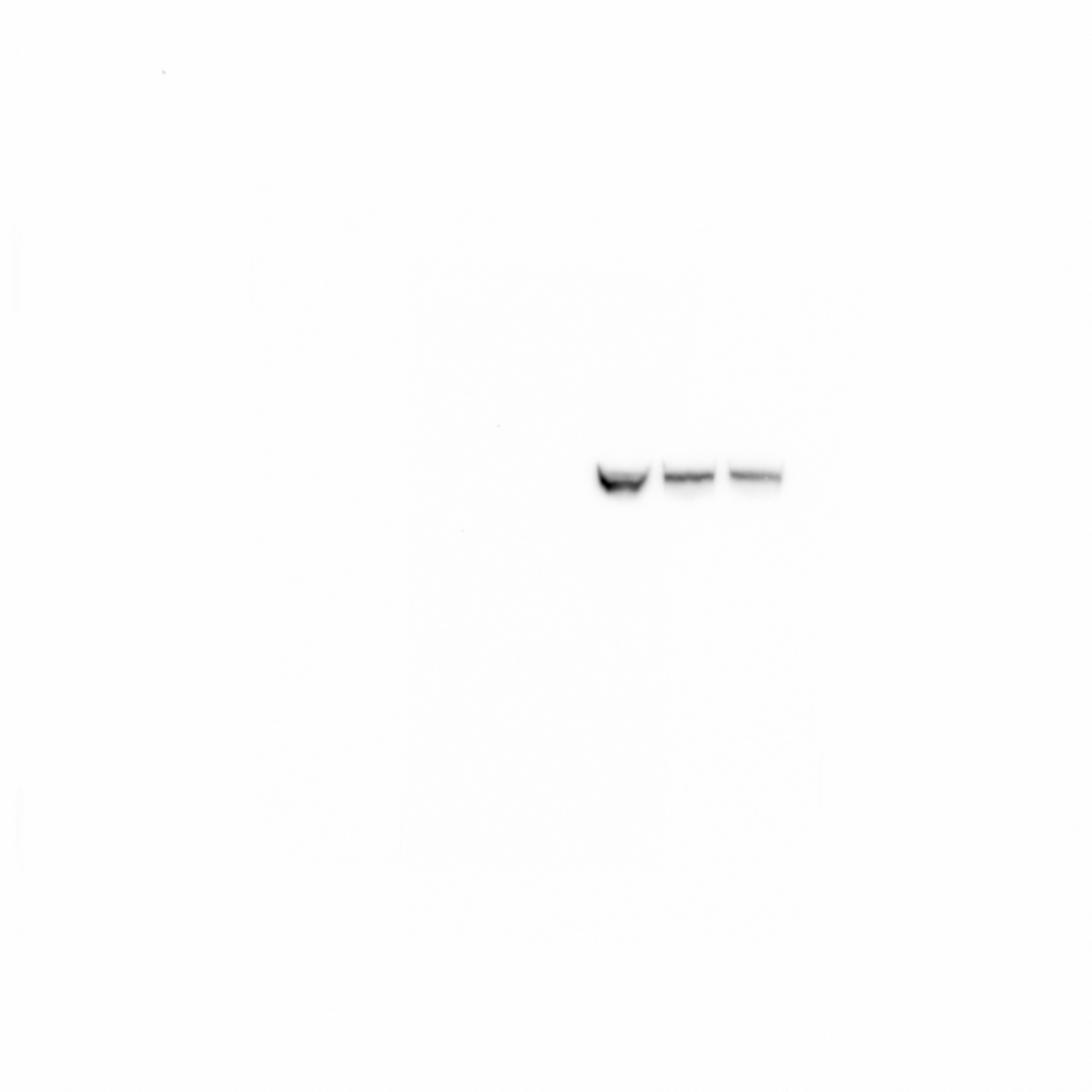

Supplement: Figure 2—source data 1. — Excel files of numbers for Figure 2A, C and D; Figure 2—figure supplement 1B. [file elife-72289-fig2-data1.zip › Figure2/Fig2_Fig_Supplement1C_source_data_Ago2.tif]

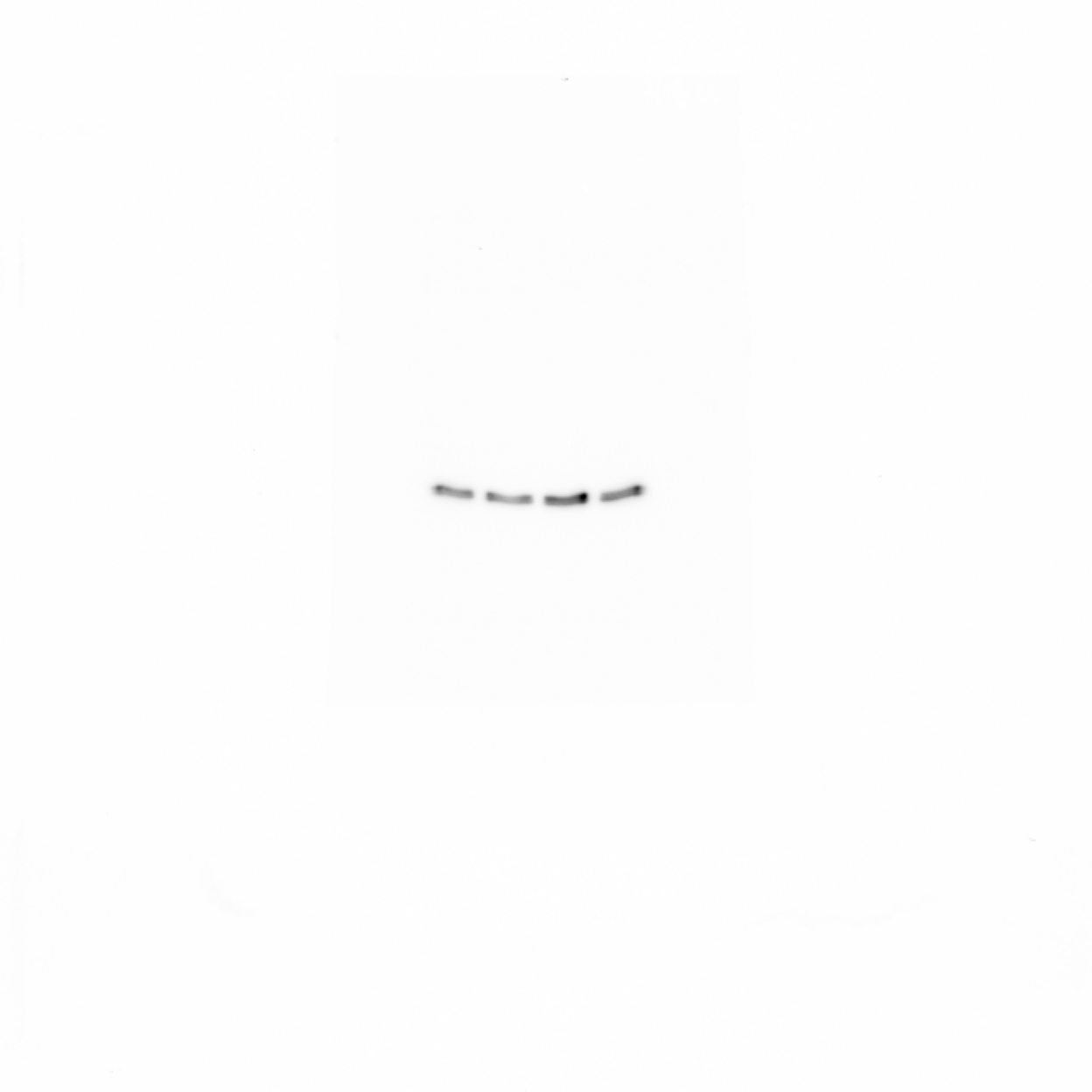

Supplement: Figure 2—source data 1. — Excel files of numbers for Figure 2A, C and D; Figure 2—figure supplement 1B. [file elife-72289-fig2-data1.zip › Figure2/Fig2B__source_data_Oct4.tif]

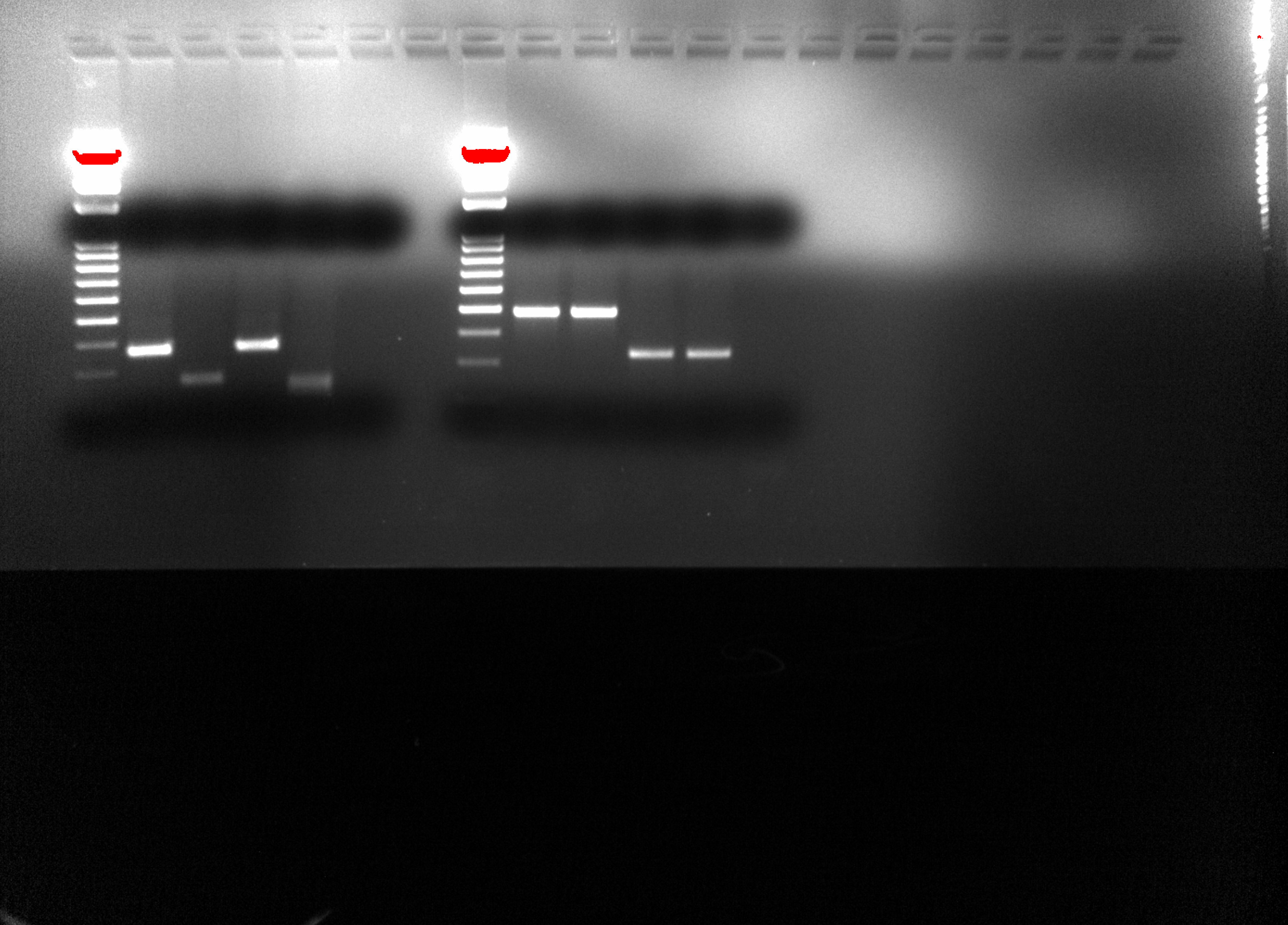

Supplement: Figure 2—source data 1. — Excel files of numbers for Figure 2A, C and D; Figure 2—figure supplement 1B. [file elife-72289-fig2-data1.zip › Figure2/Fig2_Fig_Supplement1A_source_data.tif]

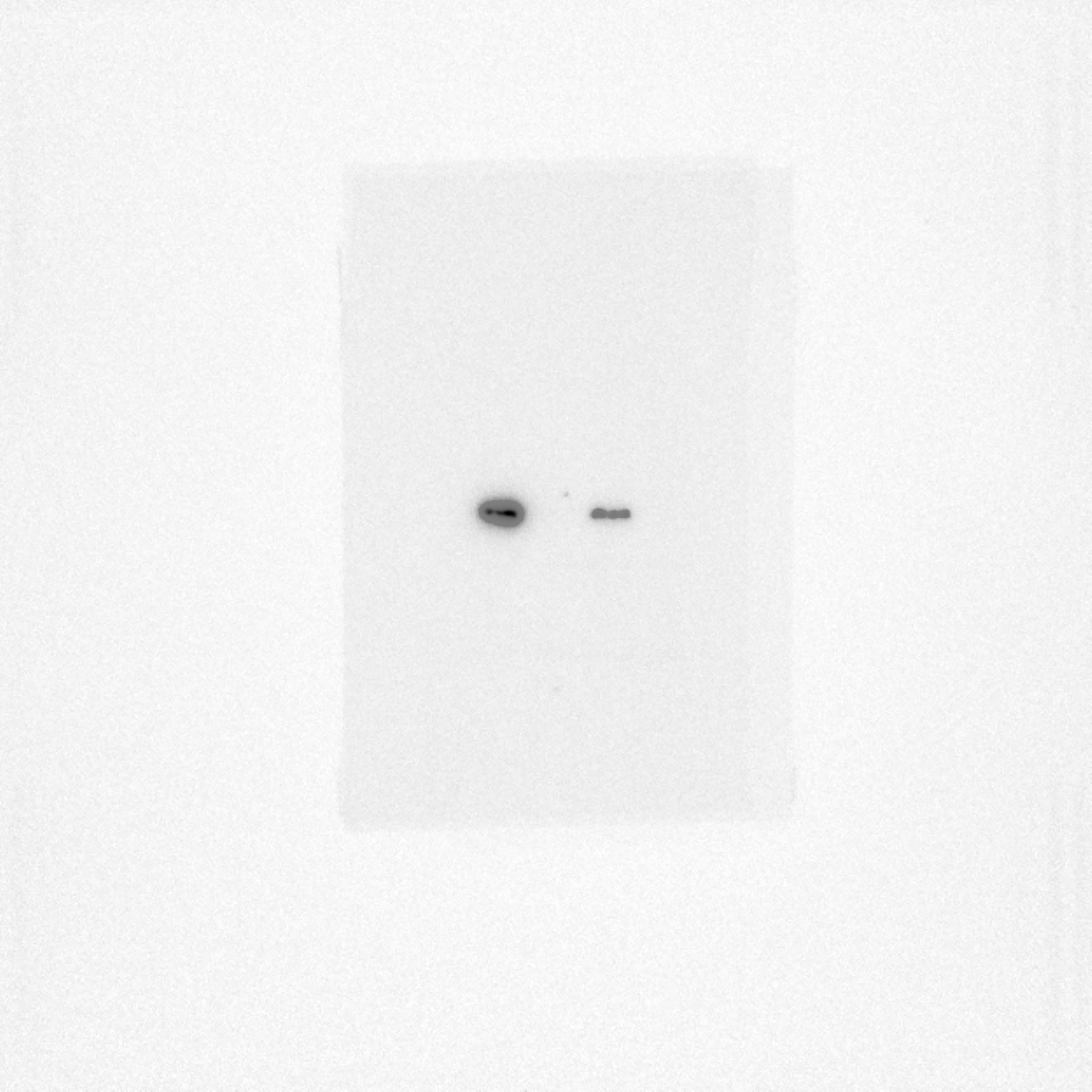

Supplement: Figure 2—source data 1. — Excel files of numbers for Figure 2A, C and D; Figure 2—figure supplement 1B. [file elife-72289-fig2-data1.zip › Figure2/Fig2E_source_data_Oct4.tif]

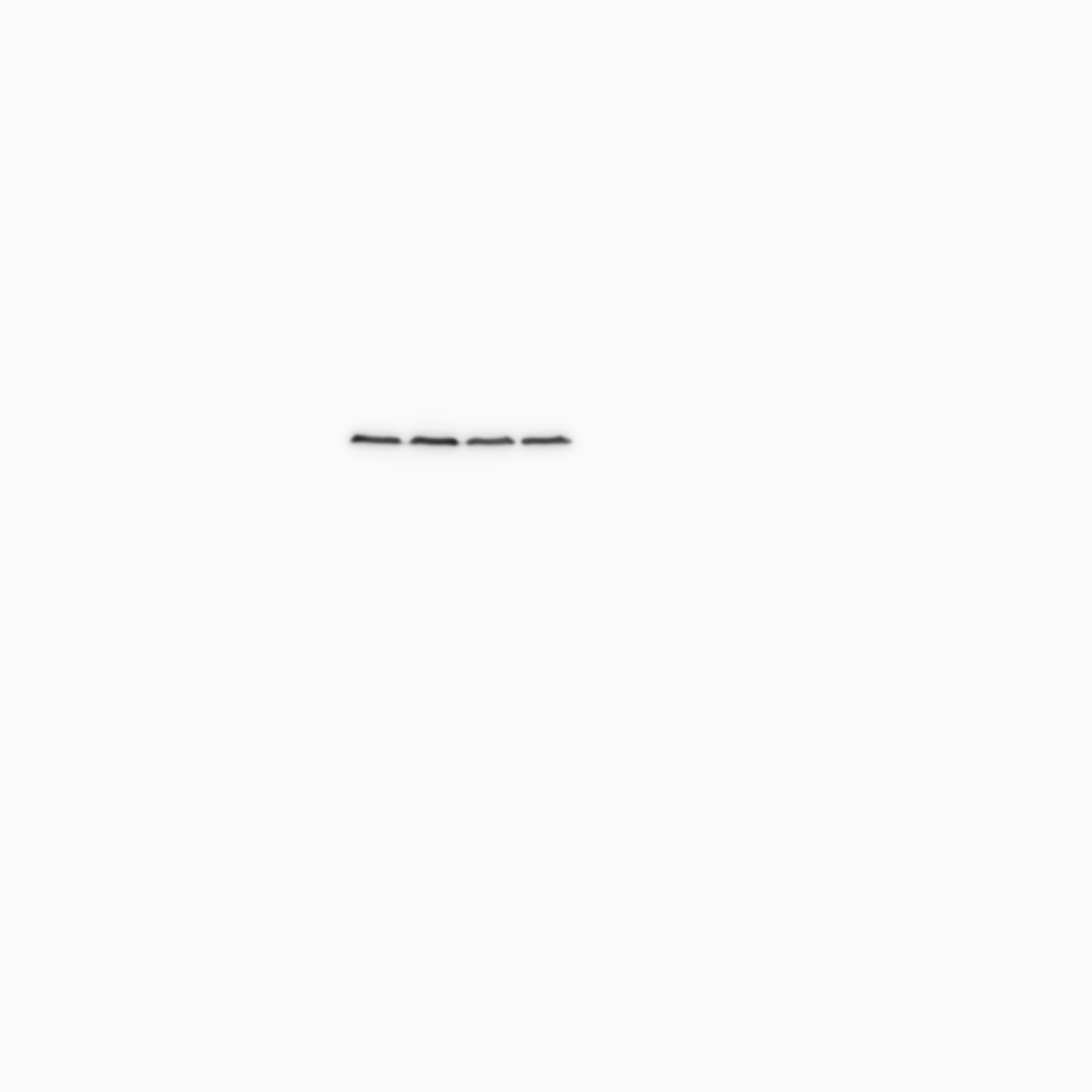

Supplement: Figure 2—source data 1. — Excel files of numbers for Figure 2A, C and D; Figure 2—figure supplement 1B. [file elife-72289-fig2-data1.zip › Figure2/Fig2E_source_data_Tubulin.tif]

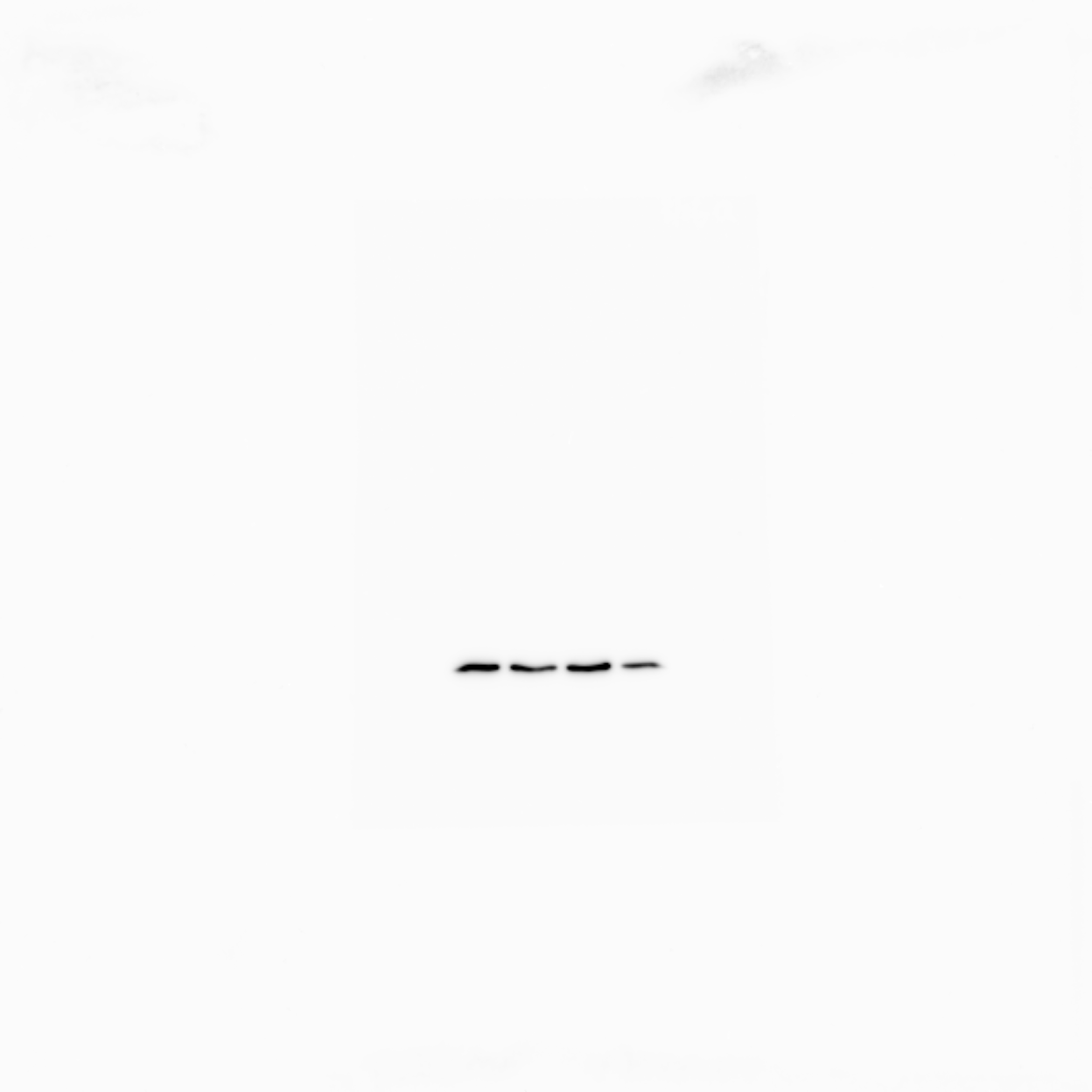

Supplement: Figure 2—source data 1. — Excel files of numbers for Figure 2A, C and D; Figure 2—figure supplement 1B. [file elife-72289-fig2-data1.zip › Figure2/Fig2B__source_data_Gapdh.tif]

Ago2

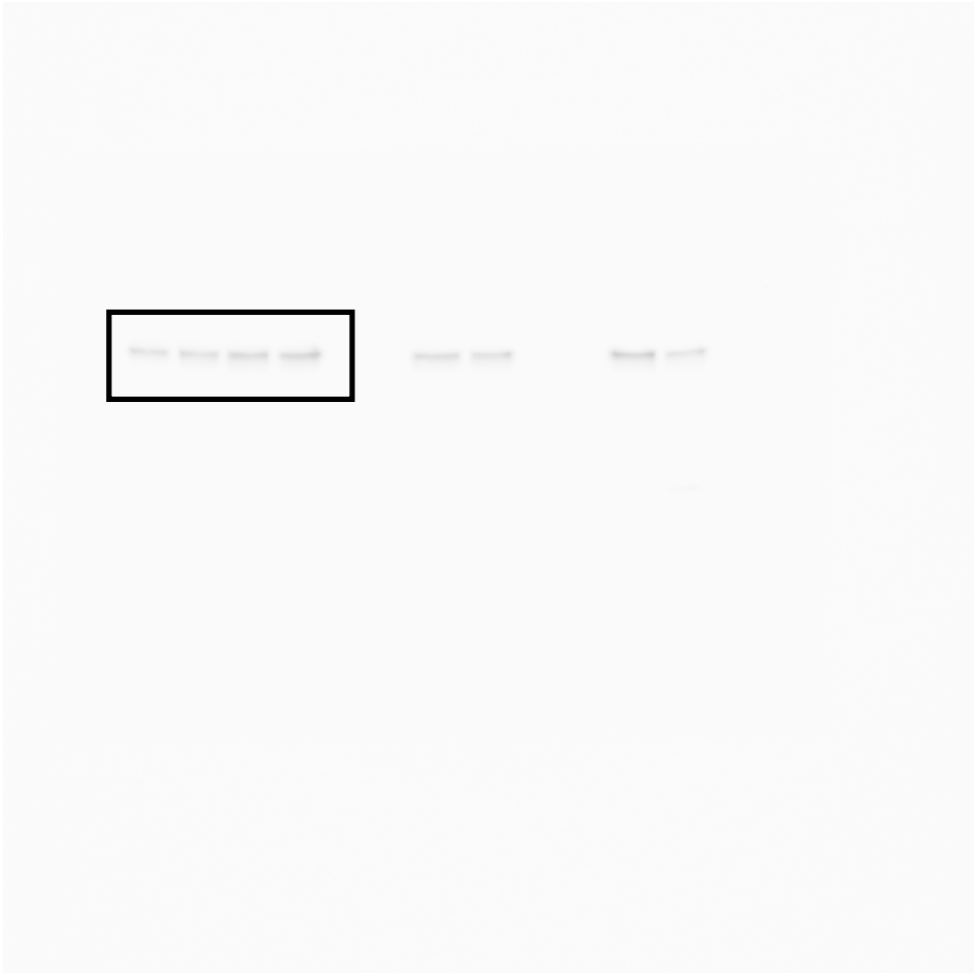

Nanog

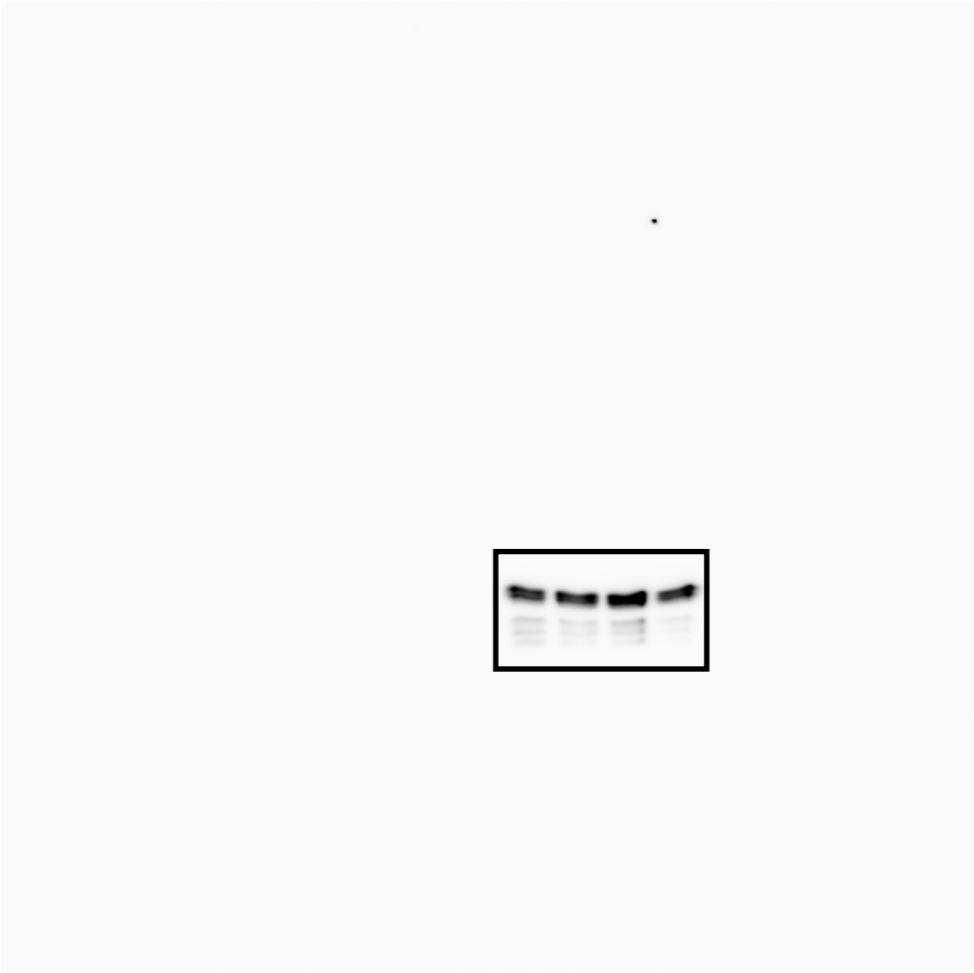

Oct4

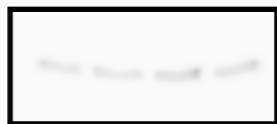

Gapdh

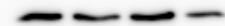

Supplement: Figure 2—source data 1. — Excel files of numbers for Figure 2A, C and D; Figure 2—figure supplement 1B. [file elife-72289-fig2-data1.zip › Figure2/uncropped_Figures/Figure2B.pdf]

Ago2

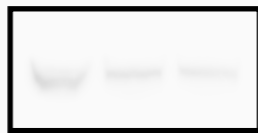

Gapdh

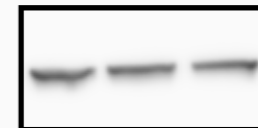

Supplement: Figure 2—source data 1. — Excel files of numbers for Figure 2A, C and D; Figure 2—figure supplement 1B. [file elife-72289-fig2-data1.zip › Figure2/uncropped_Figures/Figure2_Figure_supplement1C_uncropped.pdf]

Nanog

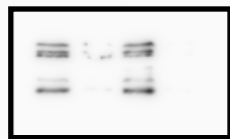

Oct4

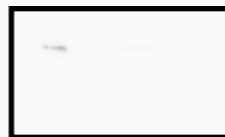

Tubulin

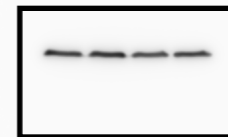

Supplement: Figure 2—source data 1. — Excel files of numbers for Figure 2A, C and D; Figure 2—figure supplement 1B. [file elife-72289-fig2-data1.zip › Figure2/uncropped_Figures/Figure2E_uncropped.pdf]

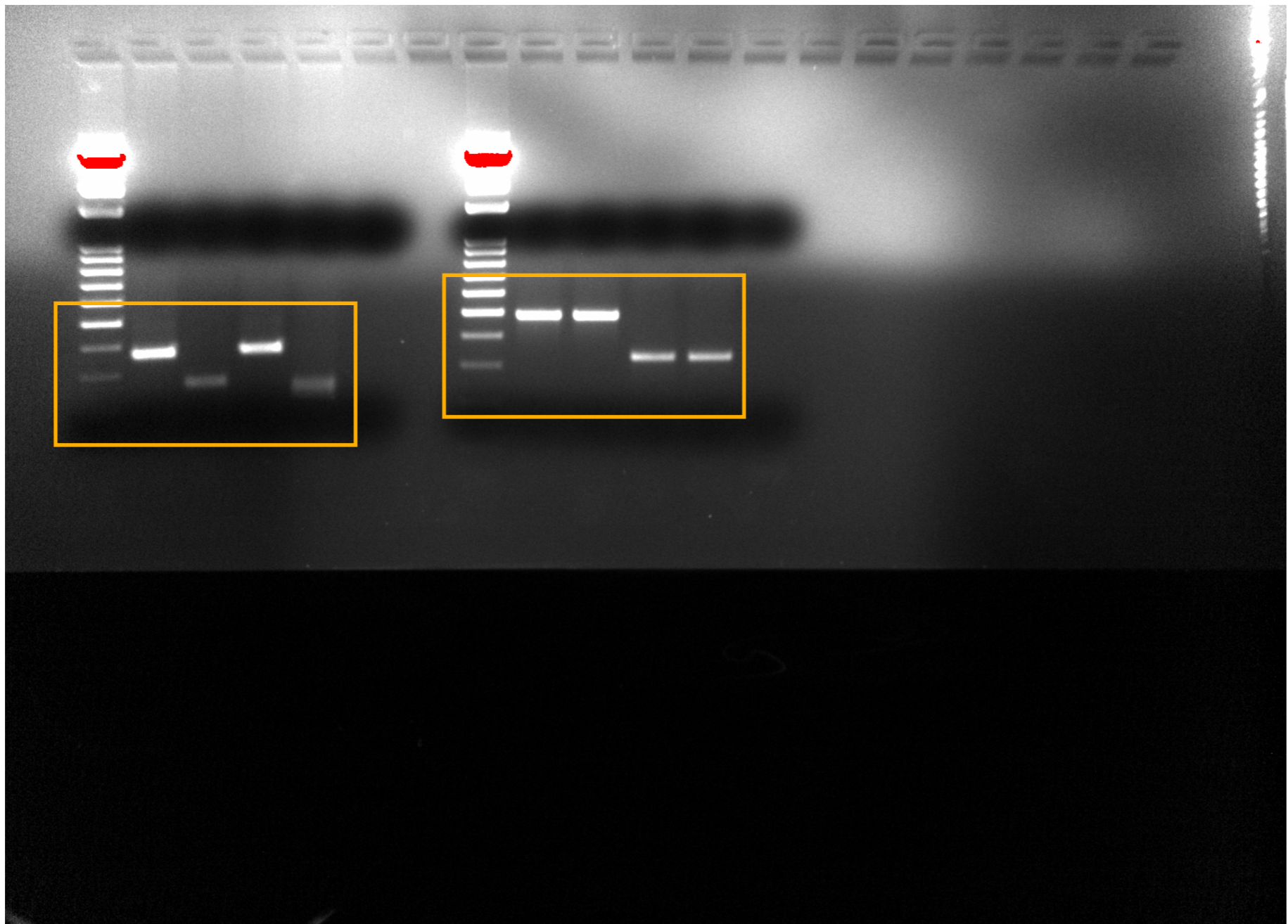

Supplement: Figure 2—source data 1. — Excel files of numbers for Figure 2A, C and D; Figure 2—figure supplement 1B. [file elife-72289-fig2-data1.zip › Figure2/uncropped_Figures/Figure2_figure_supplement1A.pdf]

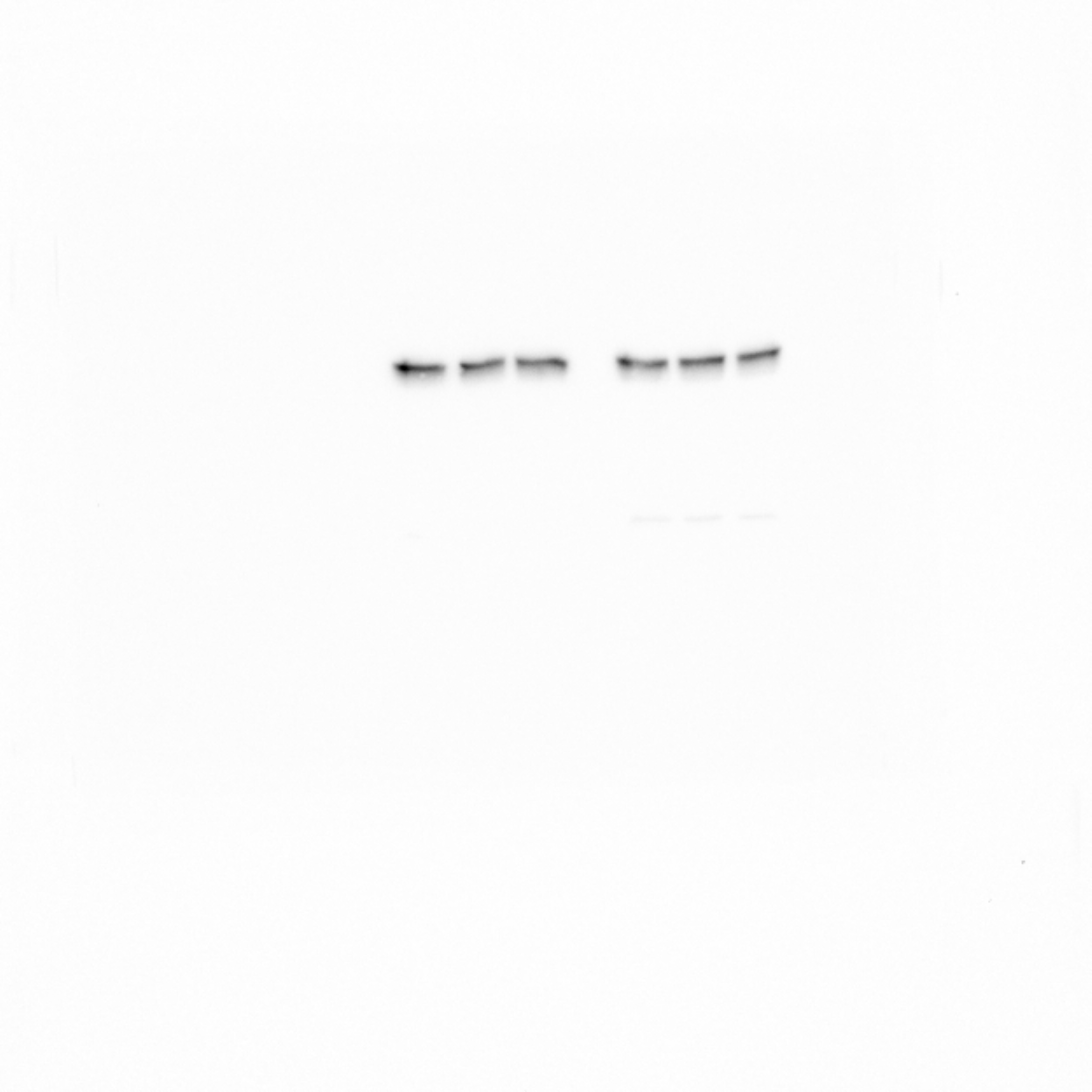

Supplement: Figure 3—source data 1. — Excel files of numbers for Figure 3D and E; Figure 3—figure supplement 1A, C. [file elife-72289-fig3-data1.zip › Figure3/Fig3_Fig_Supplement1B_source_data_Ago2.tif]

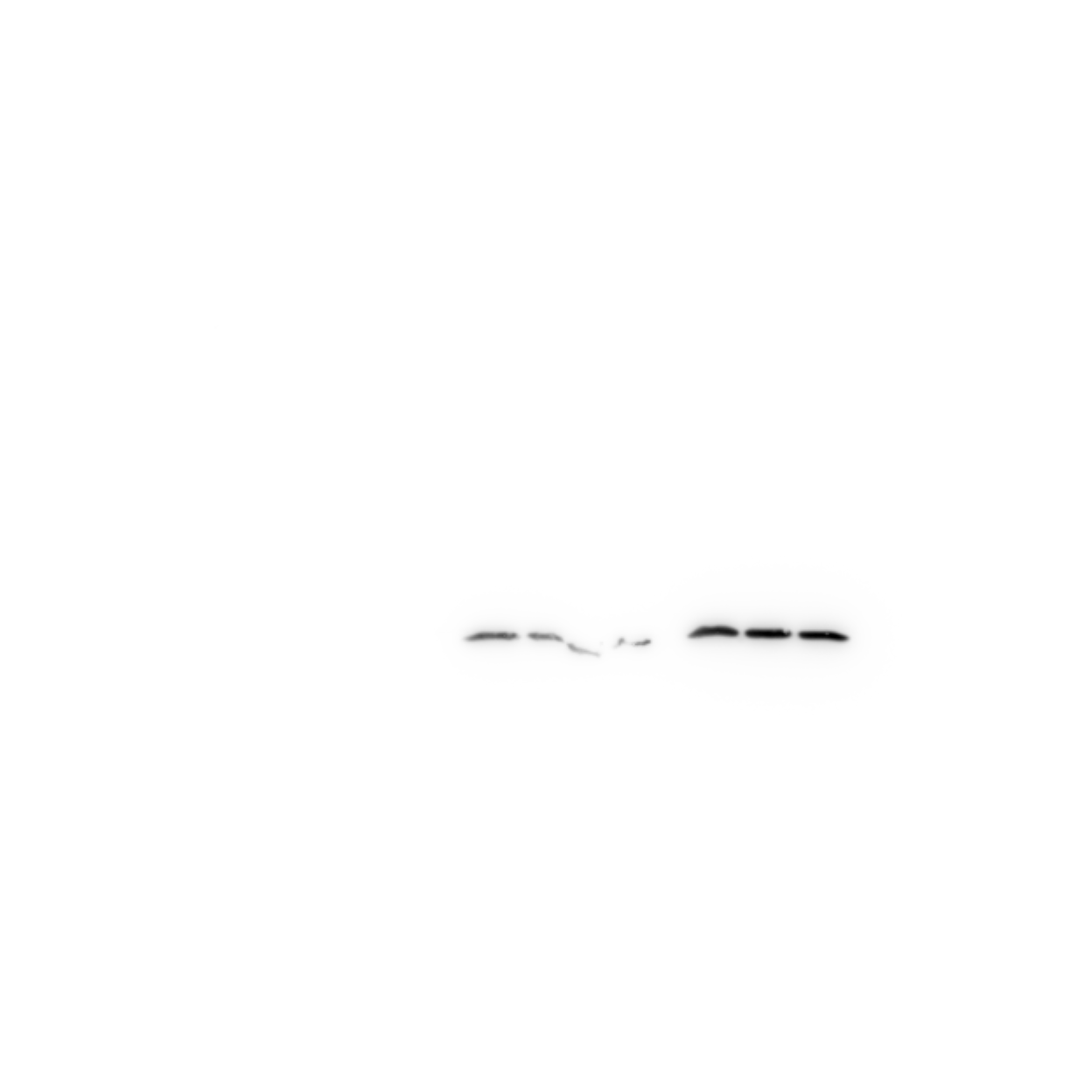

Supplement: Figure 3—source data 1. — Excel files of numbers for Figure 3D and E; Figure 3—figure supplement 1A, C. [file elife-72289-fig3-data1.zip › Figure3/Fig3_Fig_Supplement1B_source_data_Gapdh.tif]

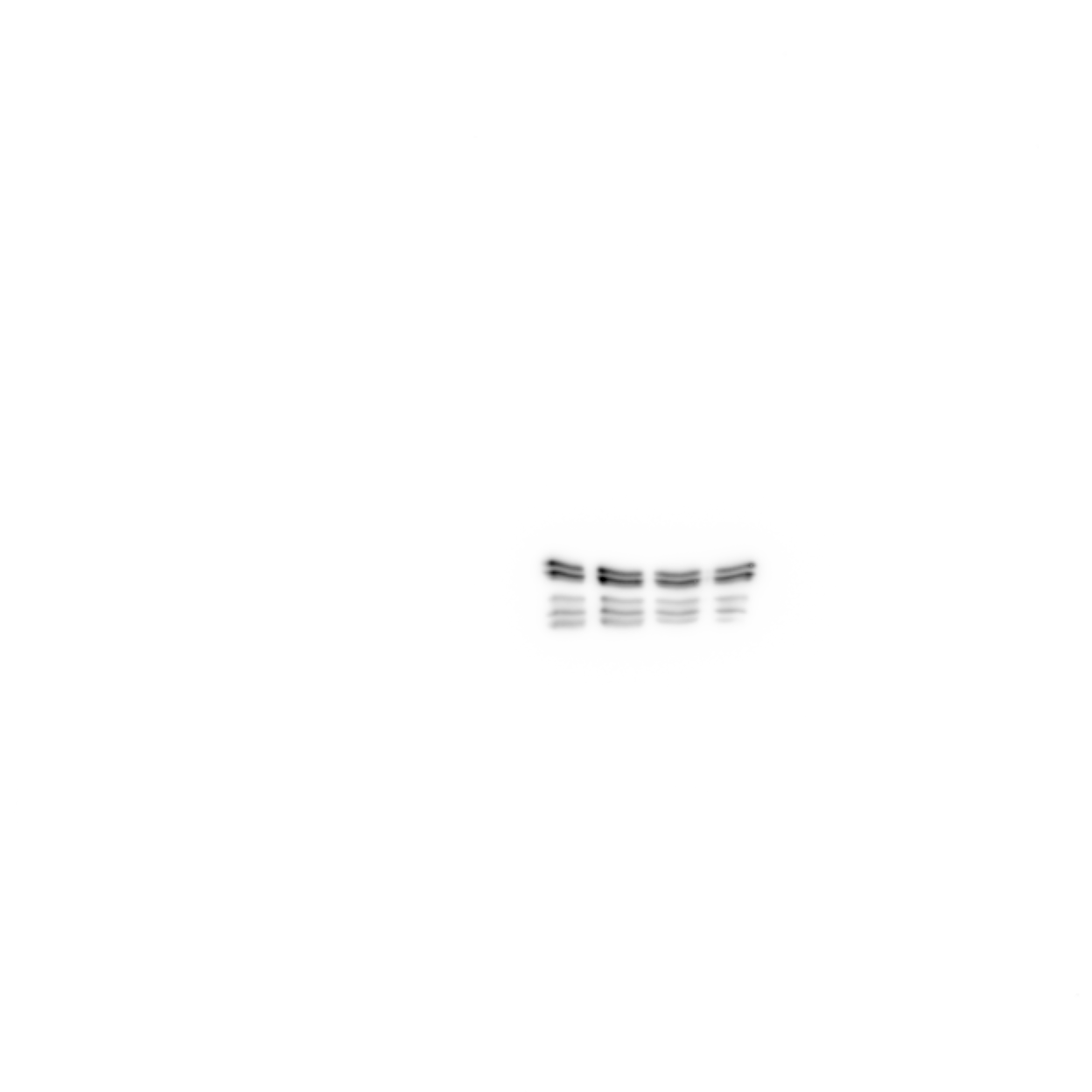

Supplement: Figure 3—source data 1. — Excel files of numbers for Figure 3D and E; Figure 3—figure supplement 1A, C. [file elife-72289-fig3-data1.zip › Figure3/Fig3C_source_data_Nanog.tif]

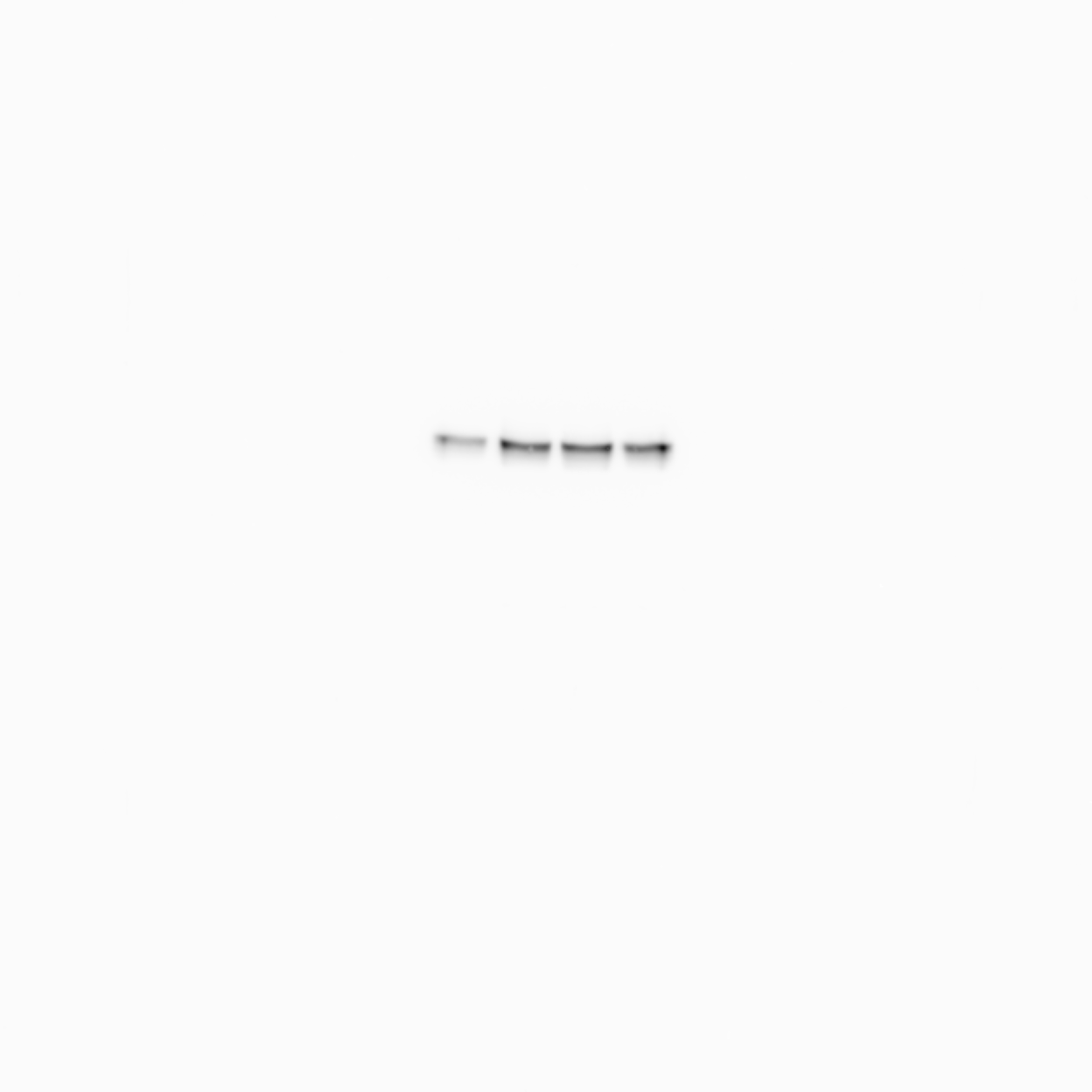

Supplement: Figure 3—source data 1. — Excel files of numbers for Figure 3D and E; Figure 3—figure supplement 1A, C. [file elife-72289-fig3-data1.zip › Figure3/Fig3C_source_data_Ago2.tif]

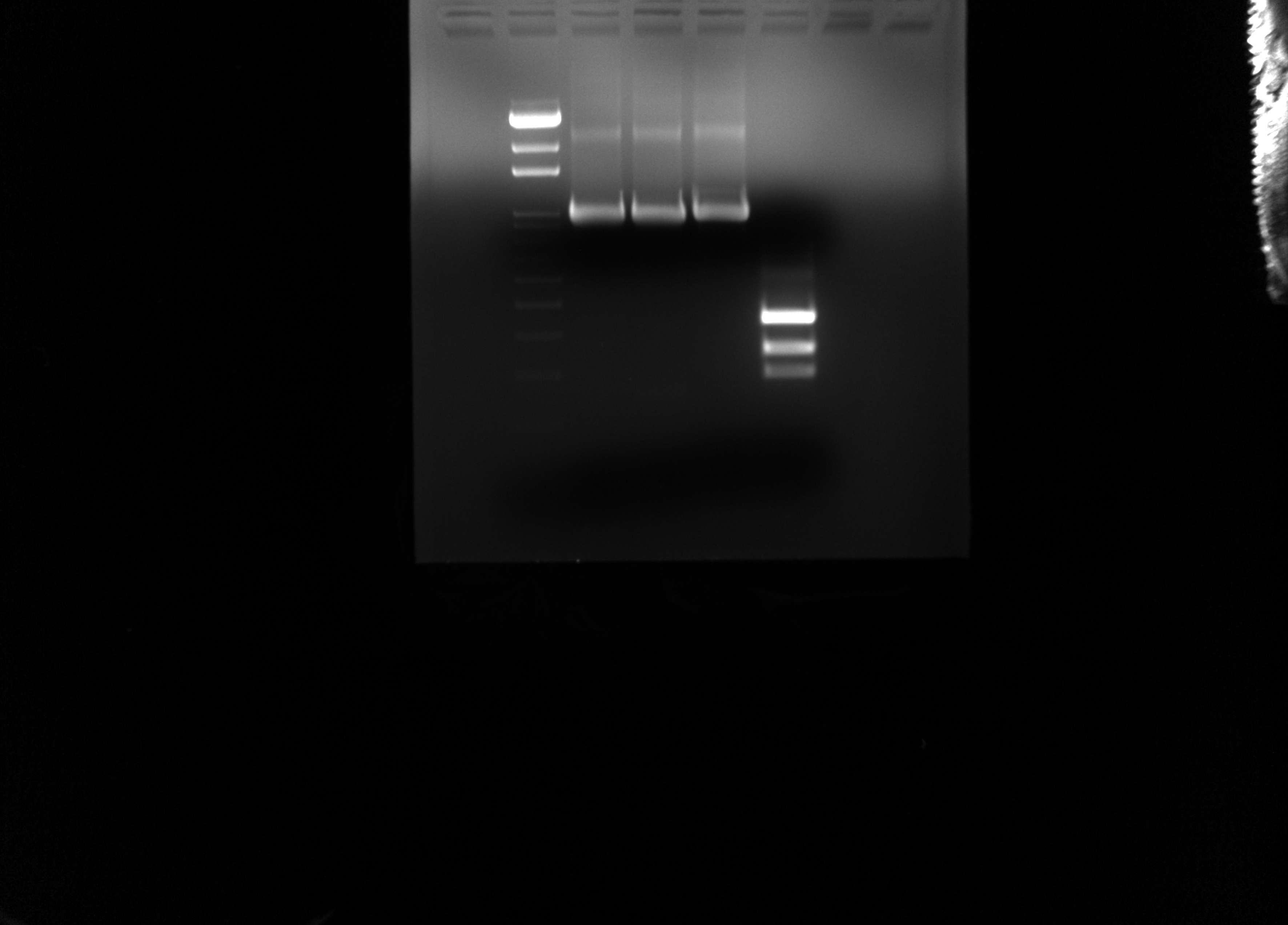

Supplement: Figure 3—source data 1. — Excel files of numbers for Figure 3D and E; Figure 3—figure supplement 1A, C. [file elife-72289-fig3-data1.zip › Figure3/Fig3B_source_data.tif]

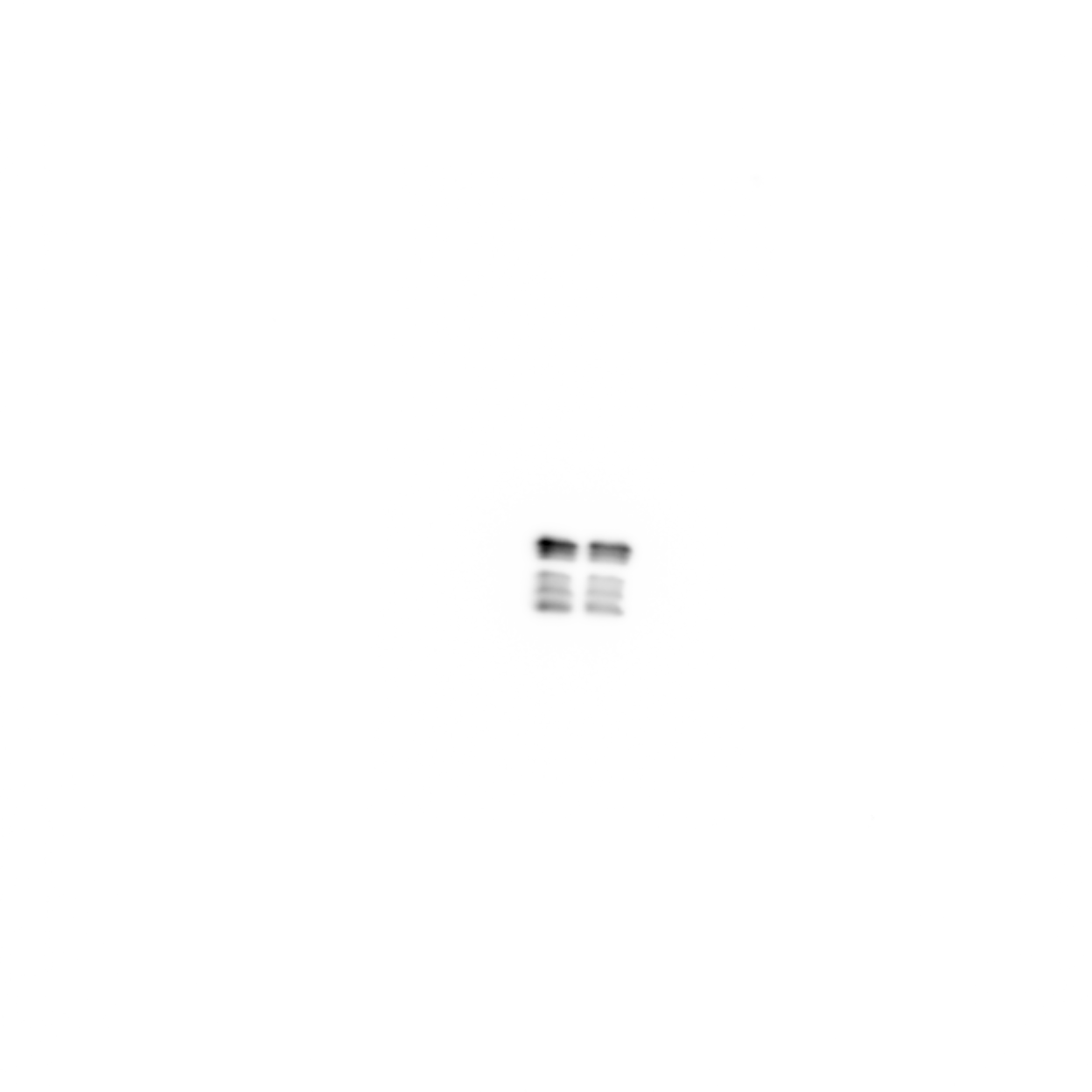

Supplement: Figure 3—source data 1. — Excel files of numbers for Figure 3D and E; Figure 3—figure supplement 1A, C. [file elife-72289-fig3-data1.zip › Figure3/Fig3F_source_data_Nanog.tif]

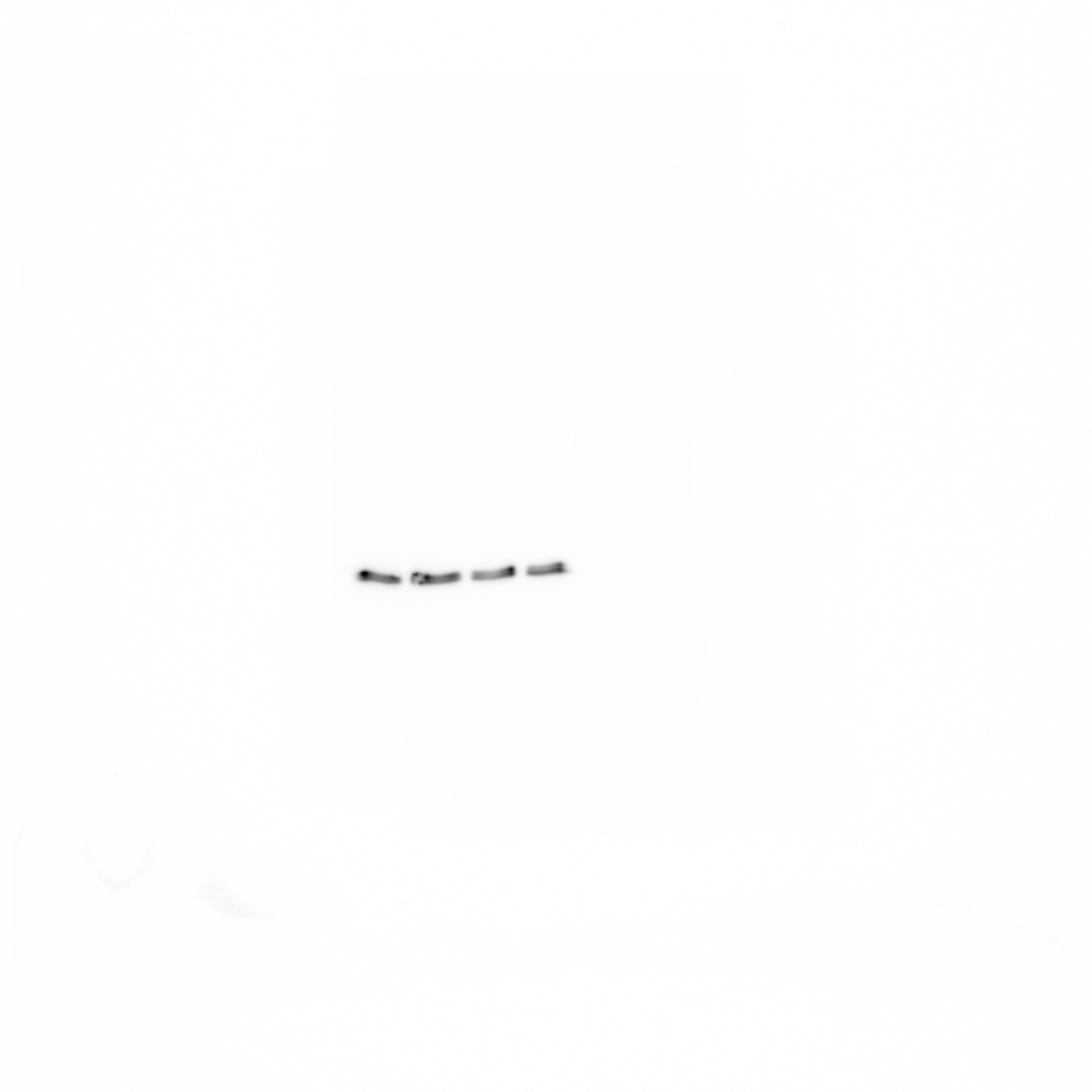

Supplement: Figure 3—source data 1. — Excel files of numbers for Figure 3D and E; Figure 3—figure supplement 1A, C. [file elife-72289-fig3-data1.zip › Figure3/Fig3C_source_data_Oct4.tif]

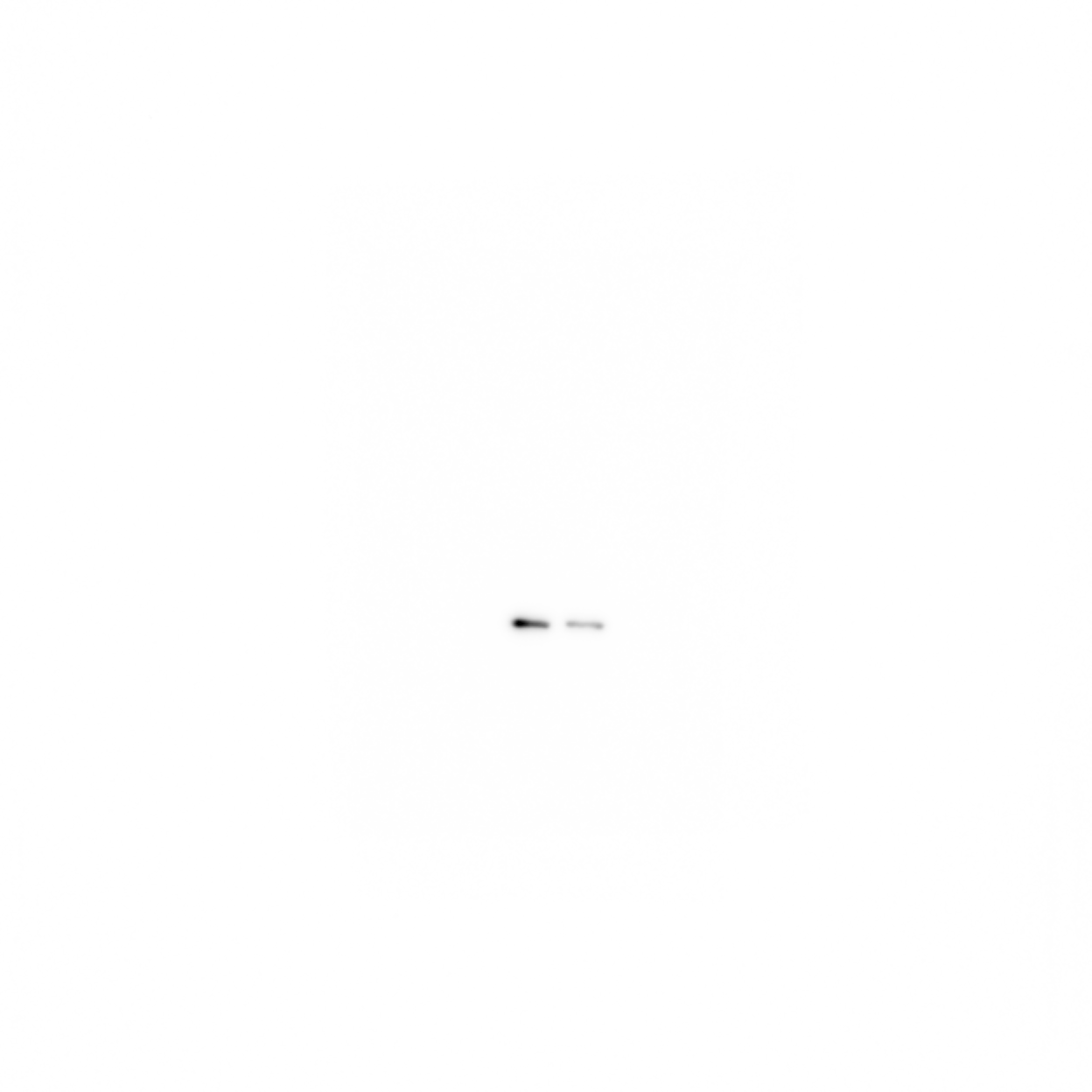

Supplement: Figure 3—source data 1. — Excel files of numbers for Figure 3D and E; Figure 3—figure supplement 1A, C. [file elife-72289-fig3-data1.zip › Figure3/Fig3F_source_data_Oct4.tif]

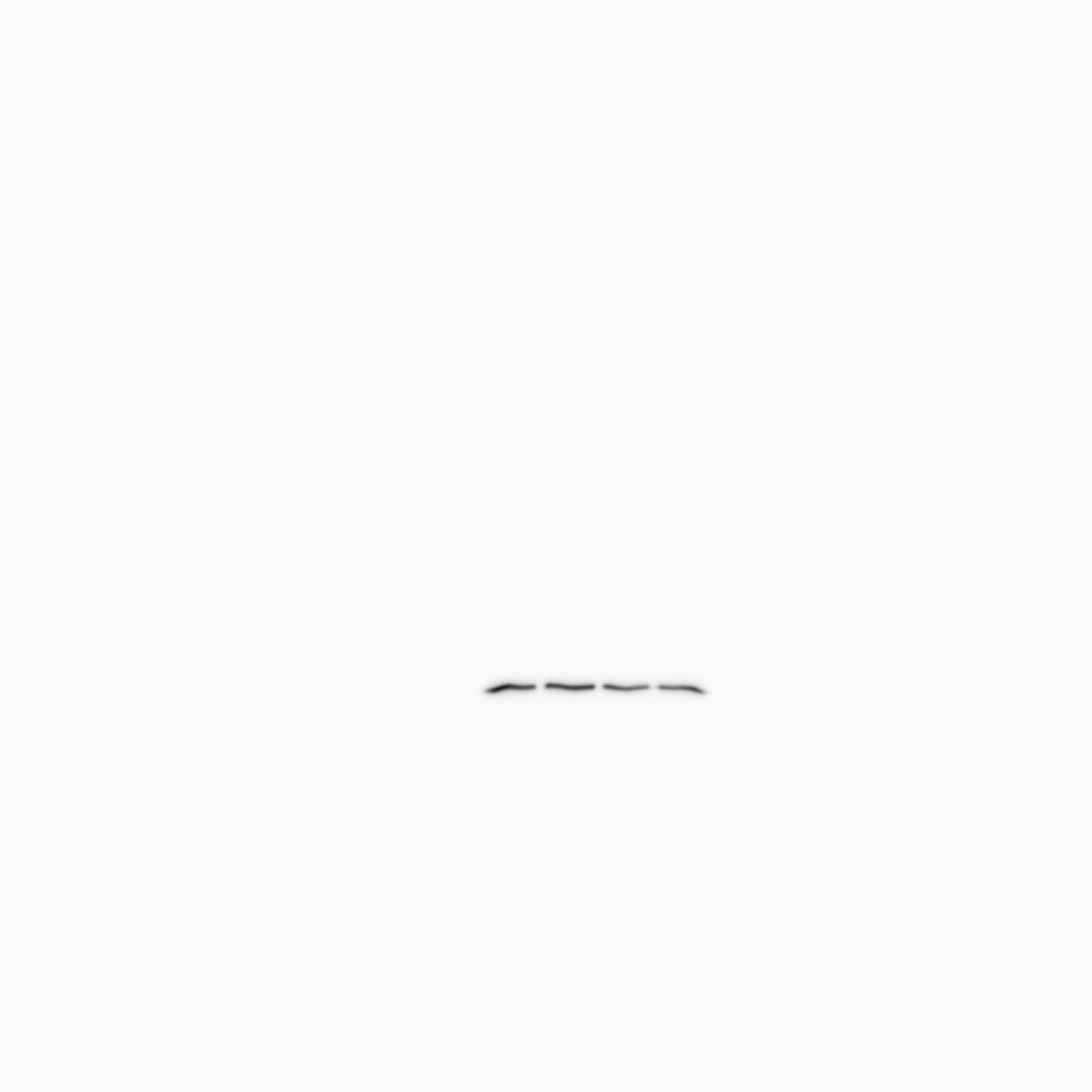

Supplement: Figure 3—source data 1. — Excel files of numbers for Figure 3D and E; Figure 3—figure supplement 1A, C. [file elife-72289-fig3-data1.zip › Figure3/Fig3C_source_data_Gapdh.tif]

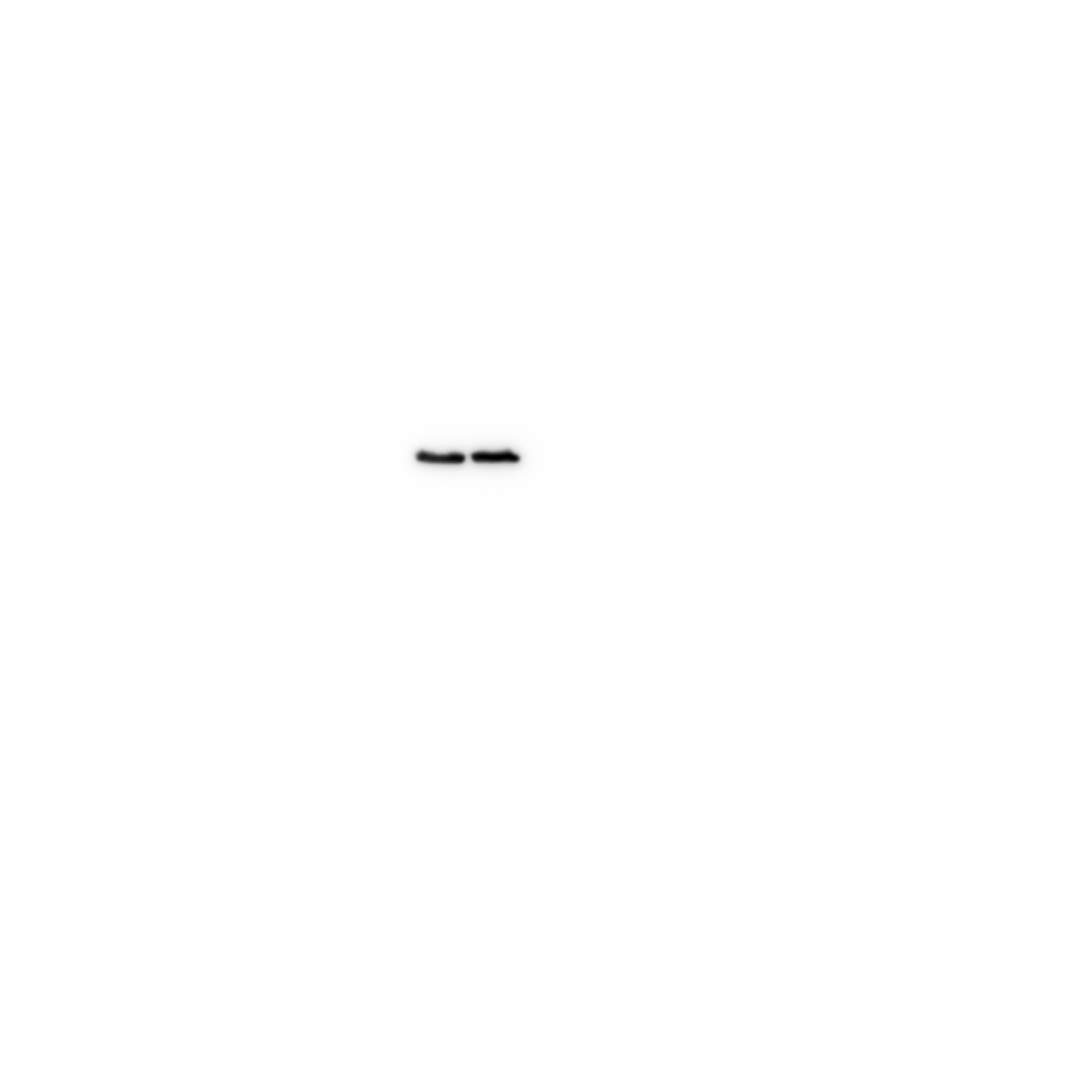

Supplement: Figure 3—source data 1. — Excel files of numbers for Figure 3D and E; Figure 3—figure supplement 1A, C. [file elife-72289-fig3-data1.zip › Figure3/Fig3F_source_data_Tubulin.tif]

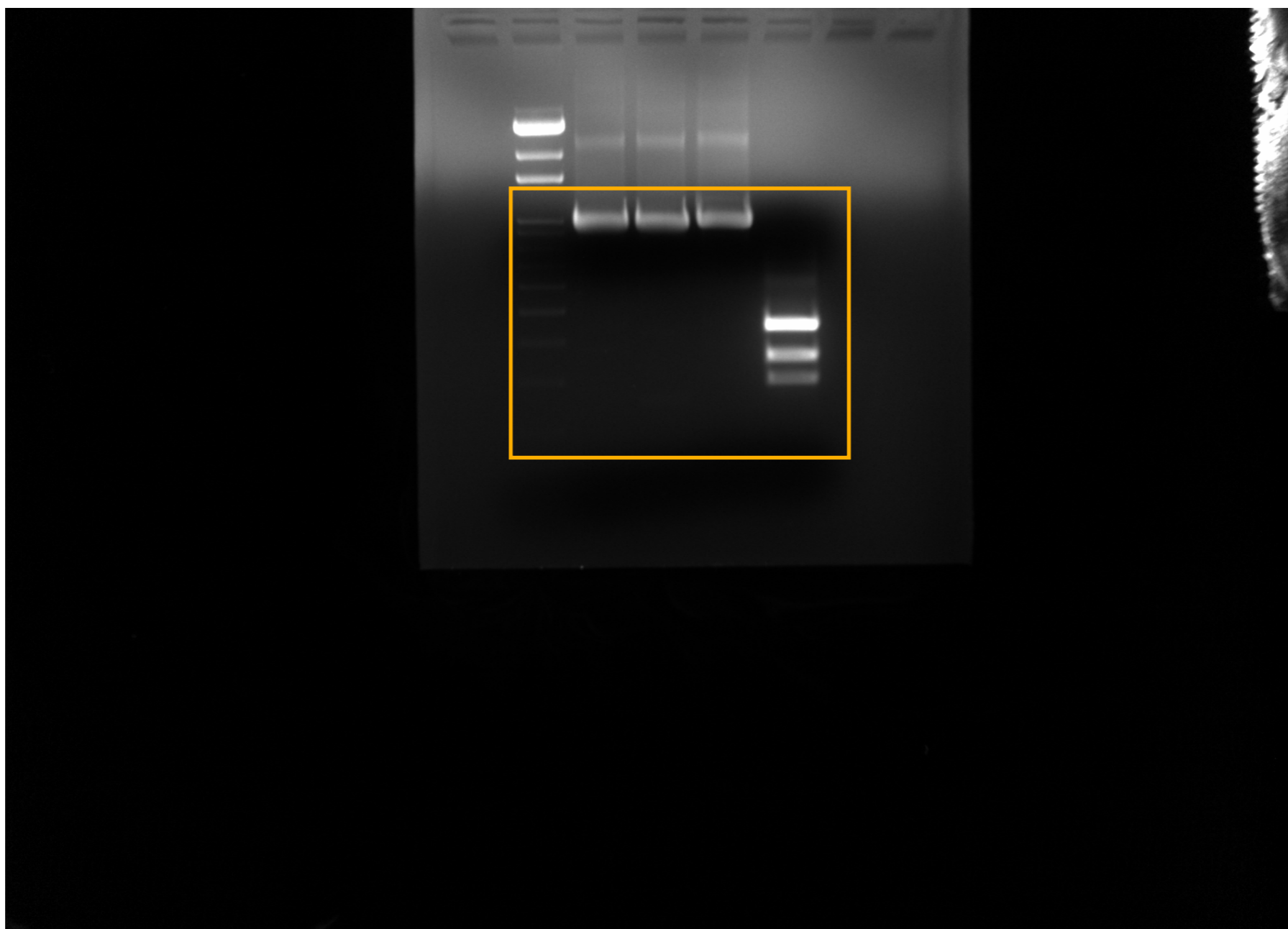

Supplement: Figure 3—source data 1. — Excel files of numbers for Figure 3D and E; Figure 3—figure supplement 1A, C. [file elife-72289-fig3-data1.zip › Figure3/Uncropped_figures/Figure3B.pdf]

Ago2

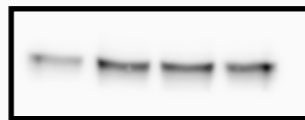

Nanog

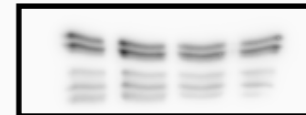

Oct4

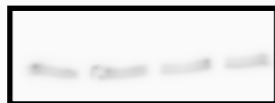

Gapdh

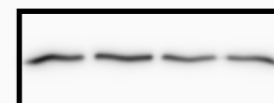

Supplement: Figure 3—source data 1. — Excel files of numbers for Figure 3D and E; Figure 3—figure supplement 1A, C. [file elife-72289-fig3-data1.zip › Figure3/Uncropped_figures/Figure3C_uncropped.pdf]

Nanog

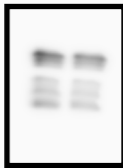

Oct4

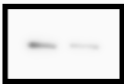

Tubulin

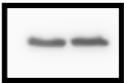

Supplement: Figure 3—source data 1. — Excel files of numbers for Figure 3D and E; Figure 3—figure supplement 1A, C. [file elife-72289-fig3-data1.zip › Figure3/Uncropped_figures/Figure3F_uncropped.pdf]

Ago2

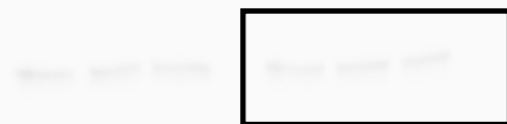

Horizontal flop

Gapdh

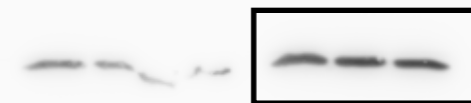

Horizontal flop

Supplement: Figure 3—source data 1. — Excel files of numbers for Figure 3D and E; Figure 3—figure supplement 1A, C. [file elife-72289-fig3-data1.zip › Figure3/Uncropped_figures/Figure3_Figure_supplement1B_uncropped.pdf]

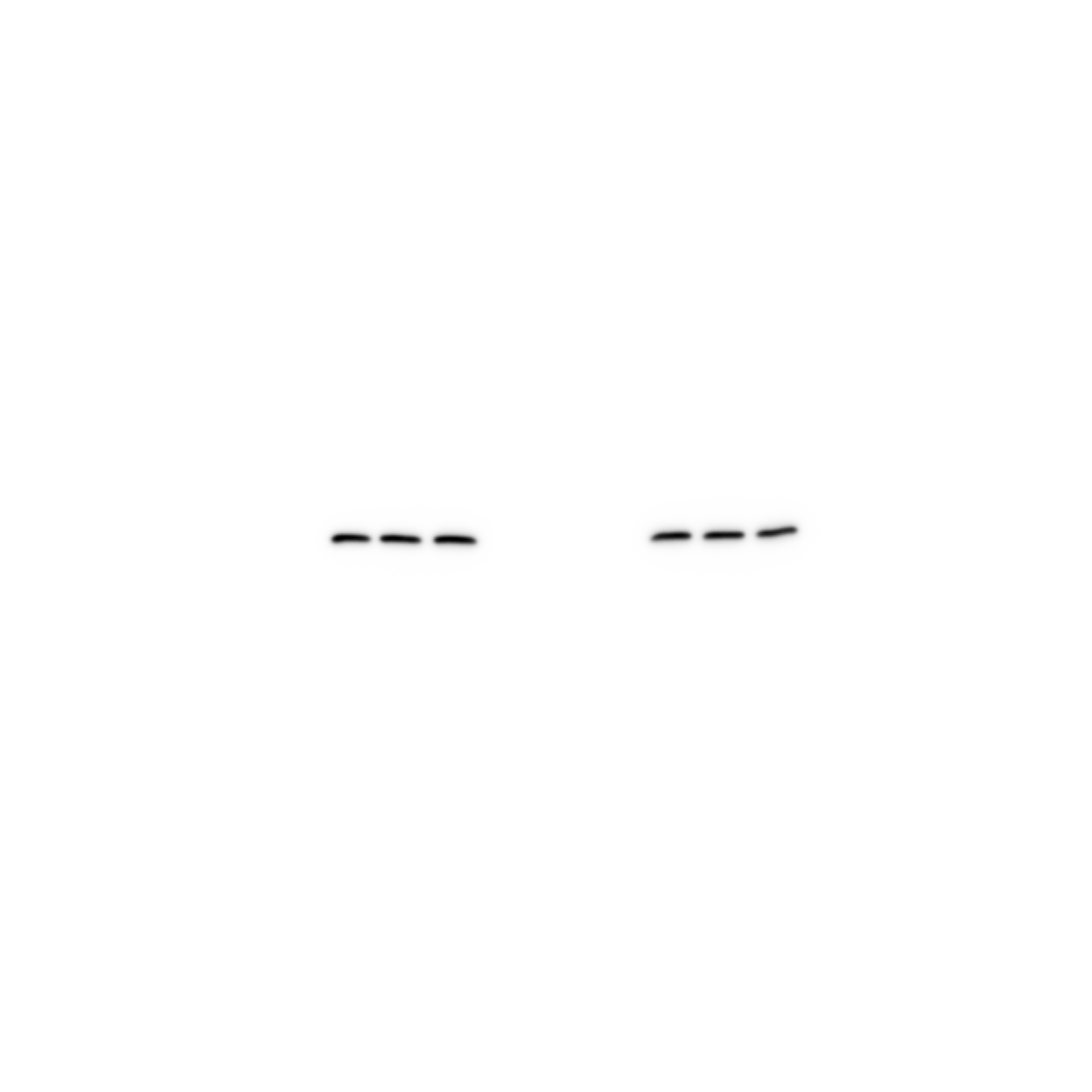

Supplement: Figure 5—source data 1. — An Excel file of numbers for Figure 5C and D. [file elife-72289-fig5-data1.zip › Figure5/Figure5B_source_data_Gapdh.tif]

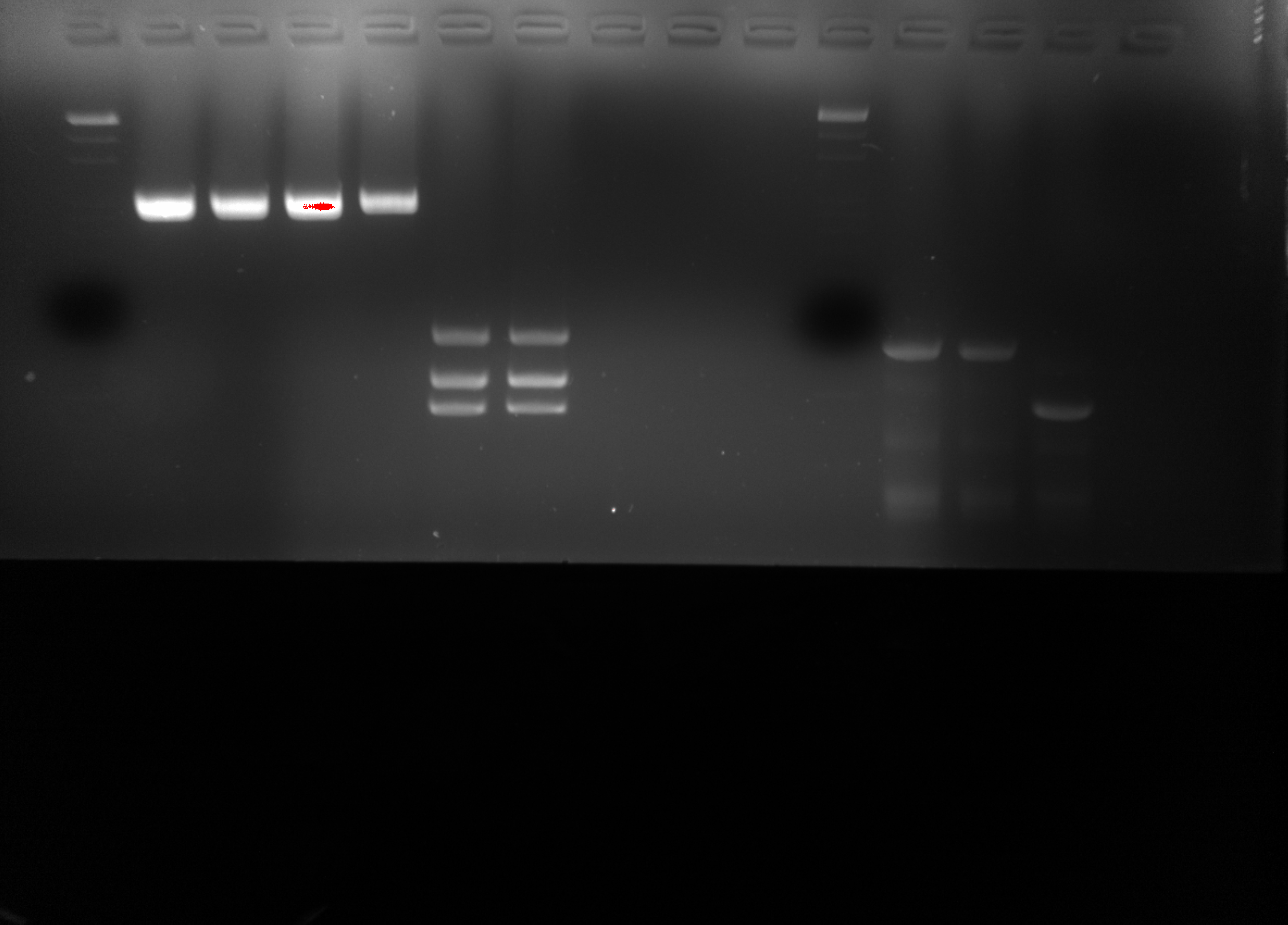

Supplement: Figure 5—source data 1. — An Excel file of numbers for Figure 5C and D. [file elife-72289-fig5-data1.zip › Figure5/Figure5_Figure_supplement1_source_data.tif]

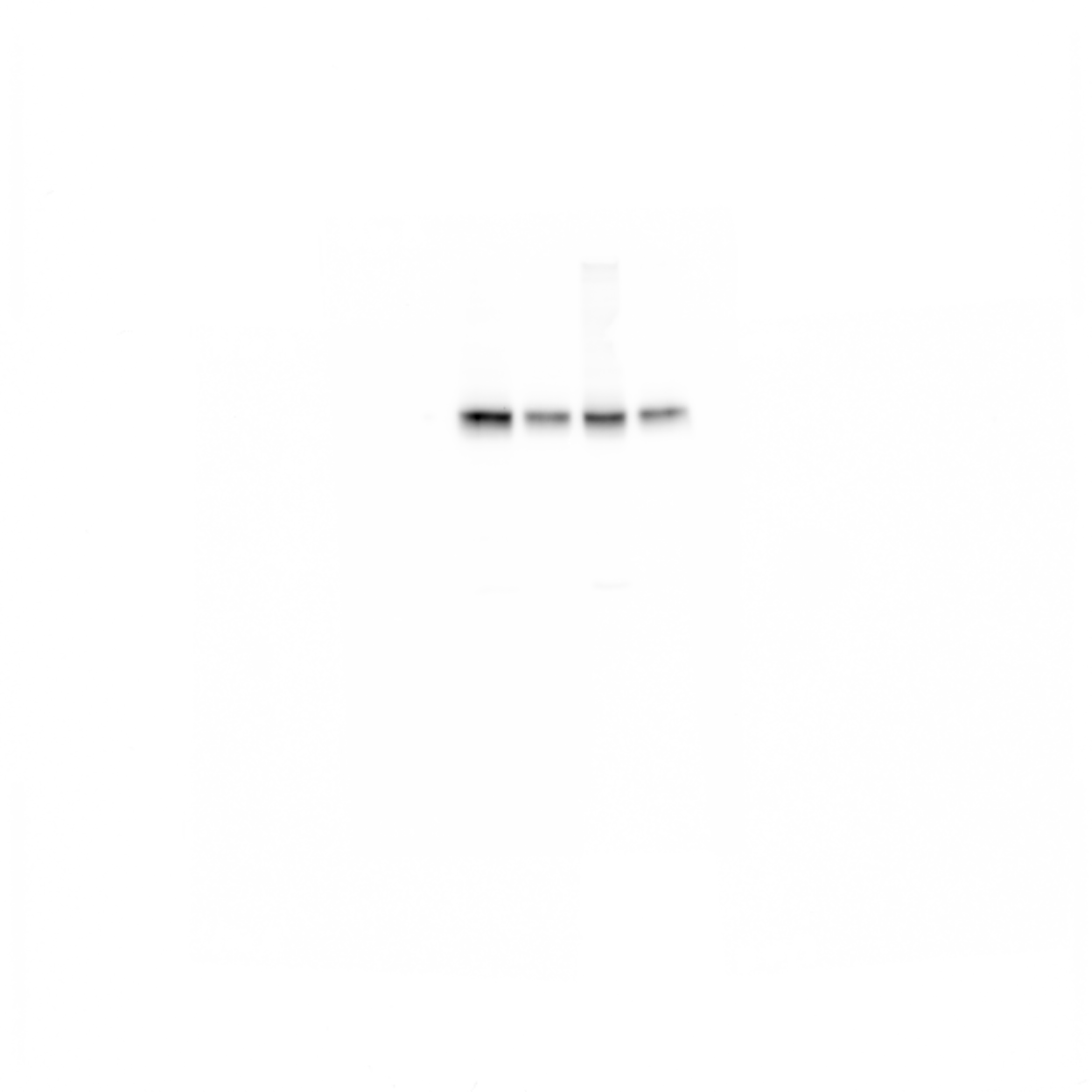

Supplement: Figure 5—source data 1. — An Excel file of numbers for Figure 5C and D. [file elife-72289-fig5-data1.zip › Figure5/Figure5A_source_data_Ago2.tif]

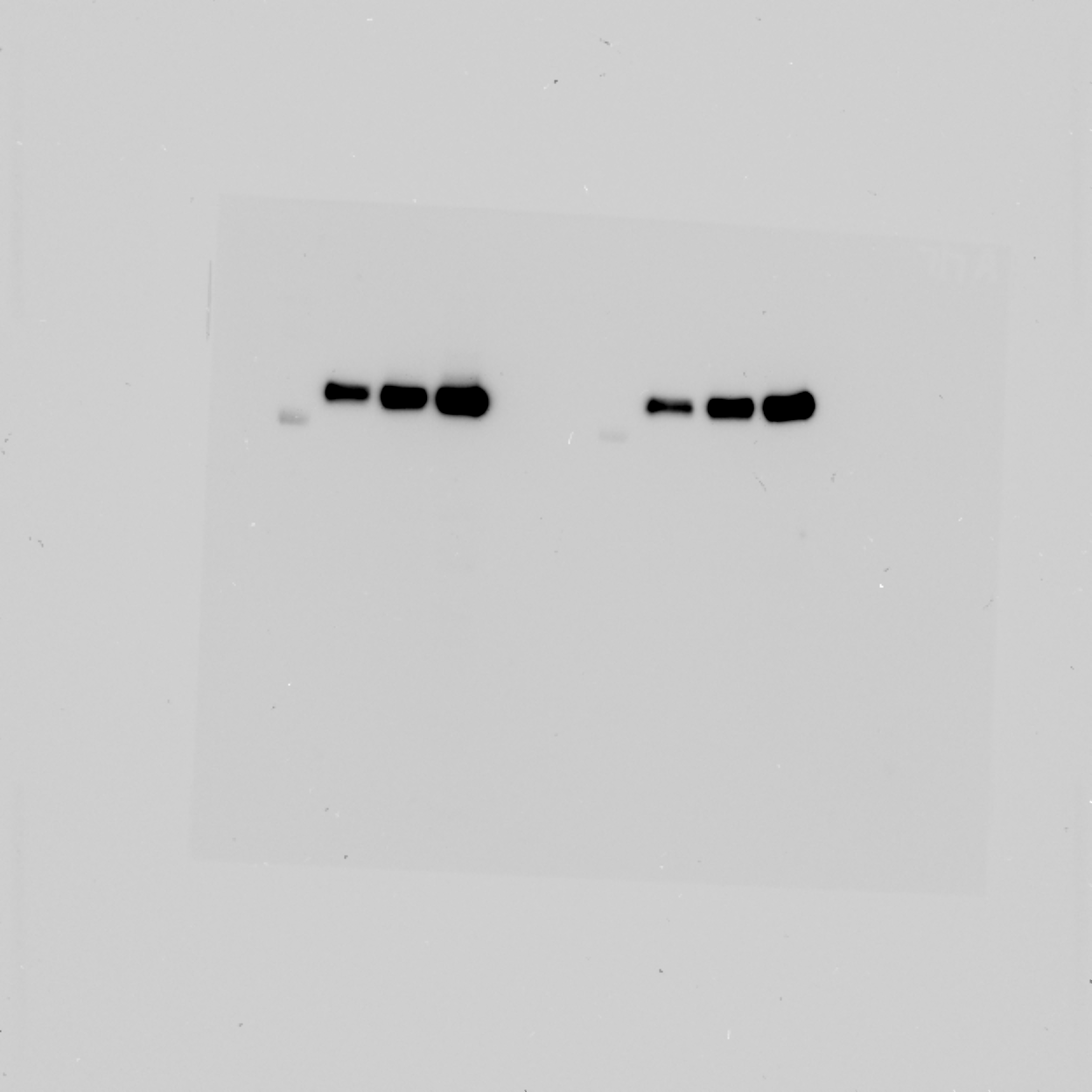

Supplement: Figure 5—source data 1. — An Excel file of numbers for Figure 5C and D. [file elife-72289-fig5-data1.zip › Figure5/Figure5B_source_data_Ago2.tif]

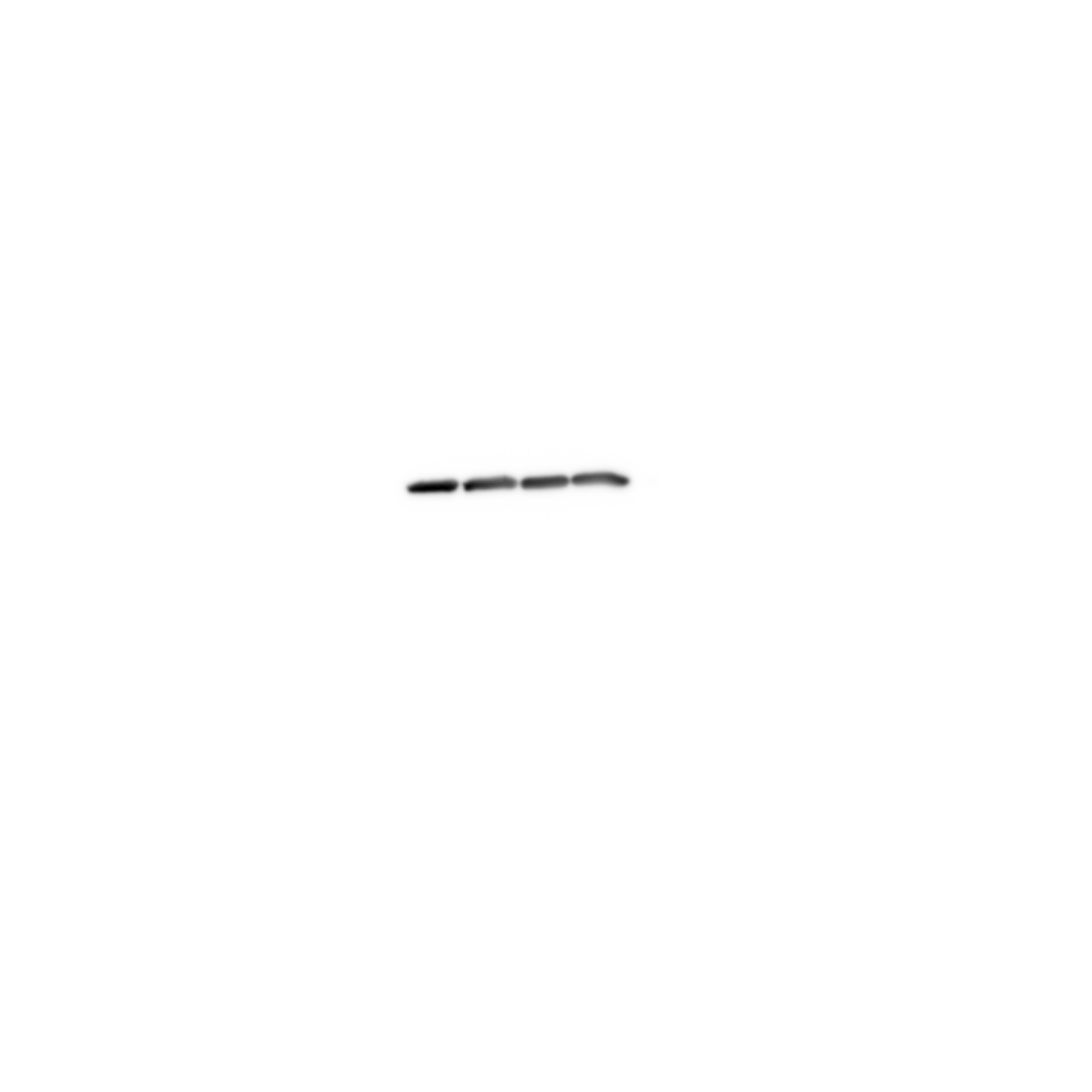

Supplement: Figure 5—source data 1. — An Excel file of numbers for Figure 5C and D. [file elife-72289-fig5-data1.zip › Figure5/Figure5A_source_data_Gapdh.tif]

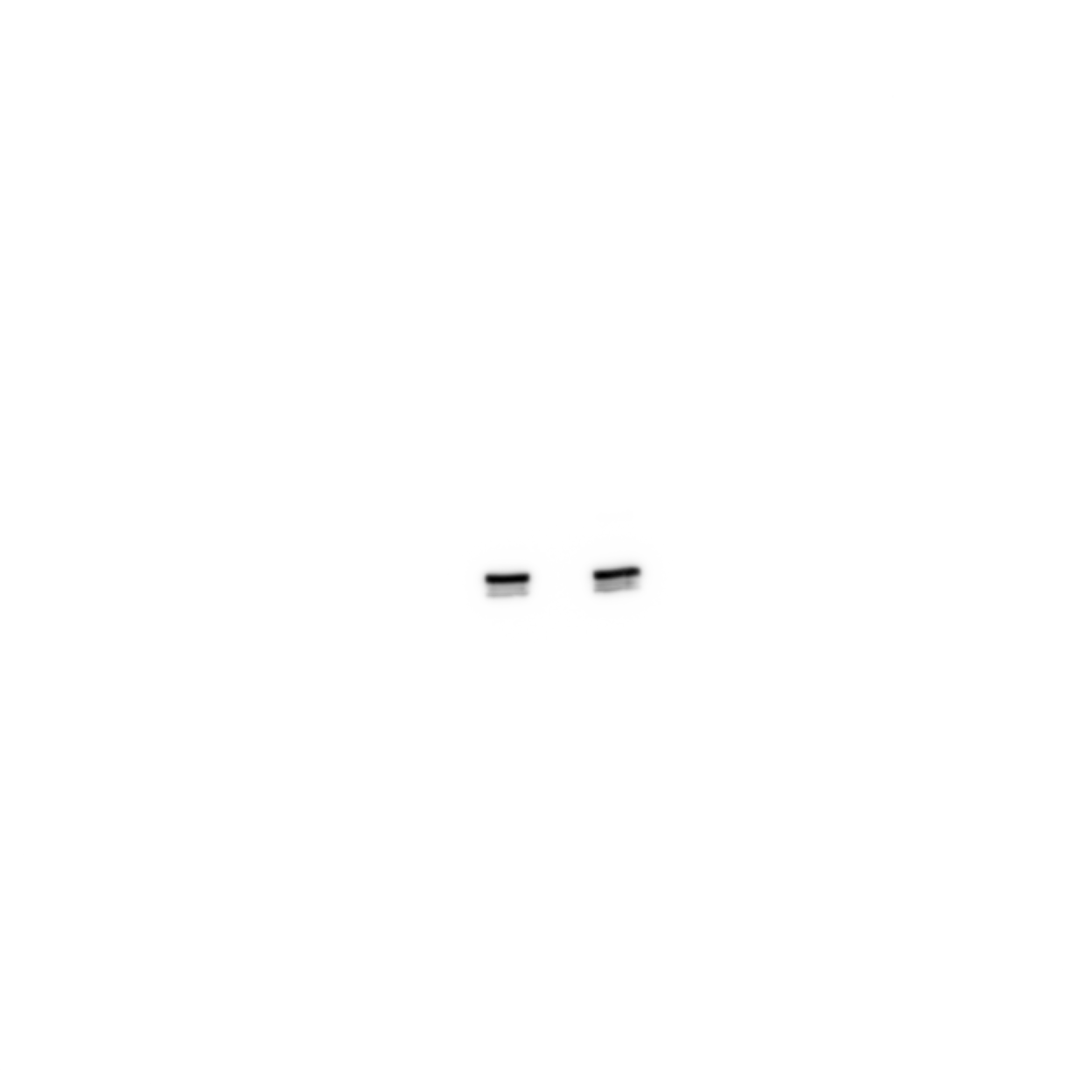

Supplement: Figure 5—source data 1. — An Excel file of numbers for Figure 5C and D. [file elife-72289-fig5-data1.zip › Figure5/Figure5A_source_data_FLAG.tif]

Figure 5 - Figure supplement 1A

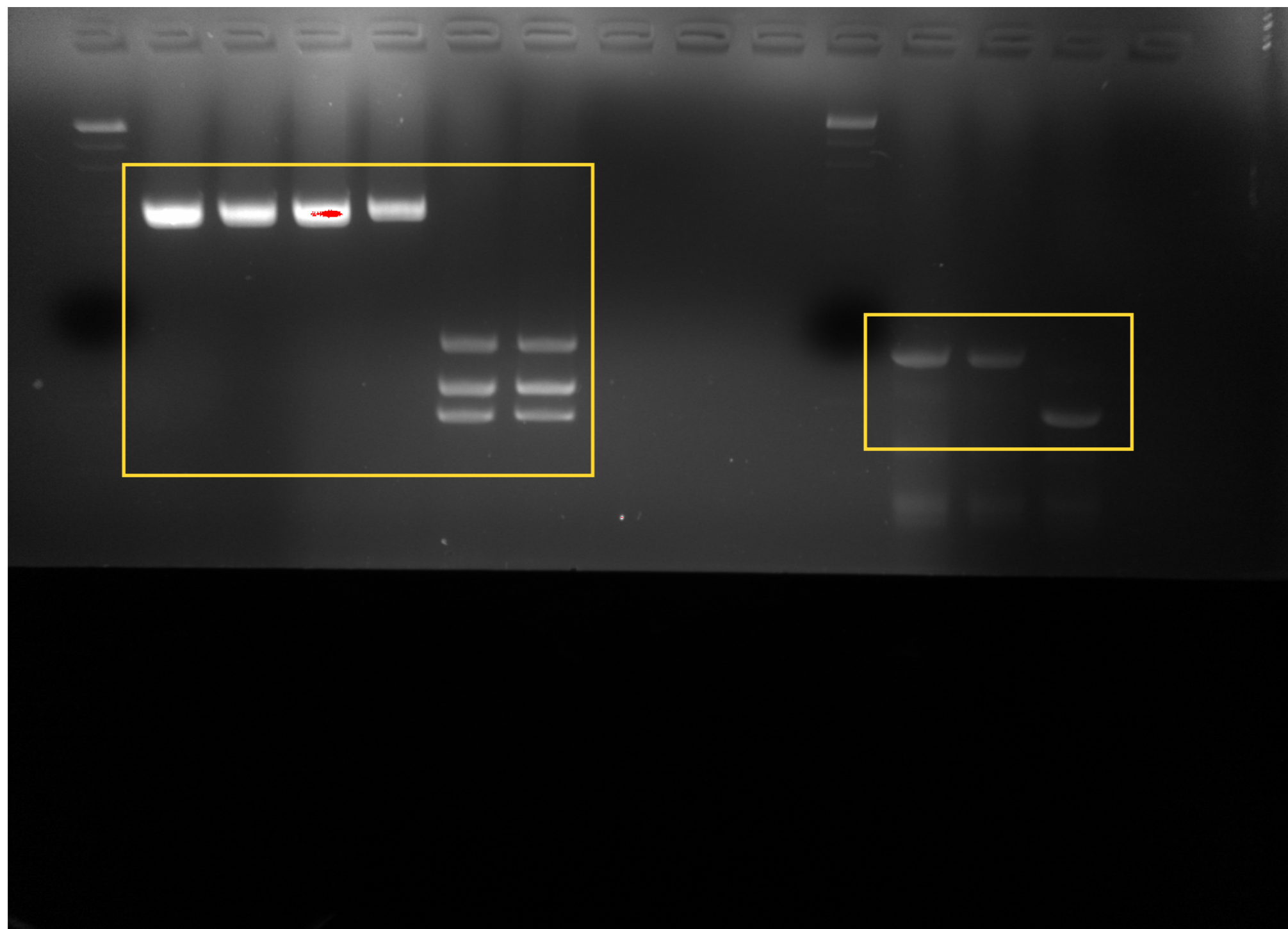

Figure 5 - Figure supplement 1B

Supplement: Figure 5—source data 1. — An Excel file of numbers for Figure 5C and D. [file elife-72289-fig5-data1.zip › Figure5/Uncropped_Figures/Figure5_Figure_supplement1_uncropped.pdf]

Ago2

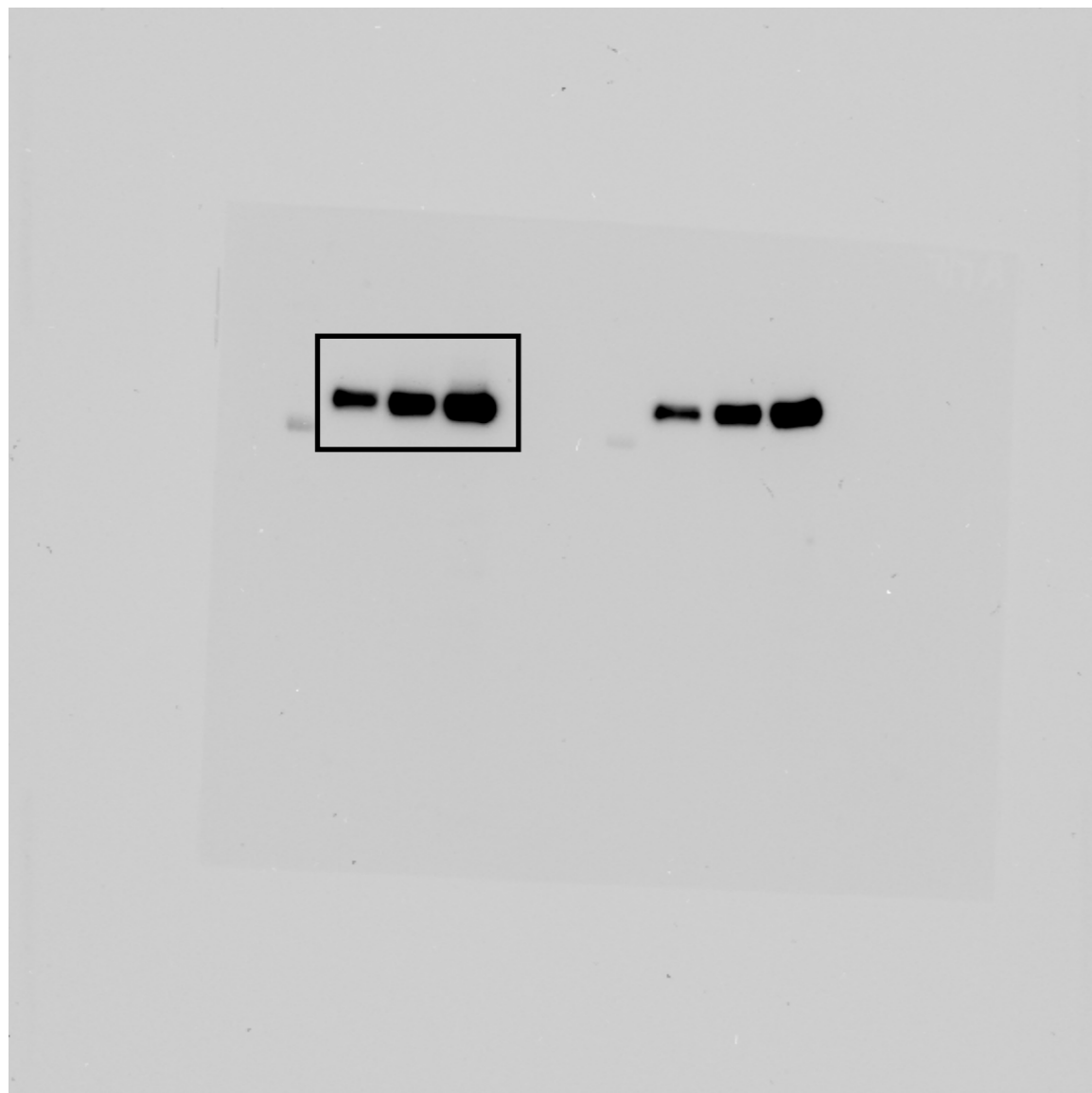

Gapdh

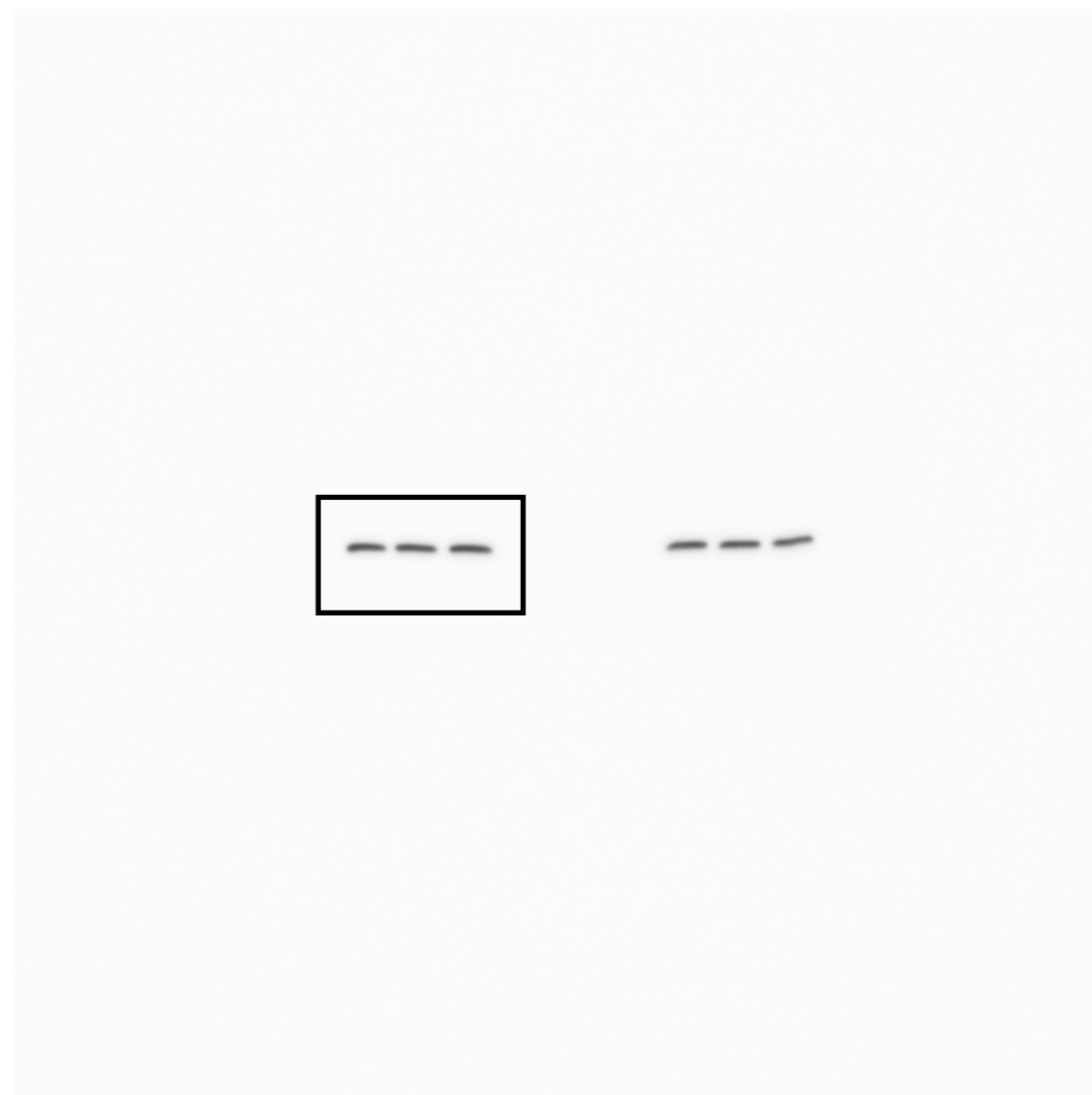

Supplement: Figure 5—source data 1. — An Excel file of numbers for Figure 5C and D. [file elife-72289-fig5-data1.zip › Figure5/Uncropped_Figures/Figure5B_uncropped.pdf]

anti-FLAG

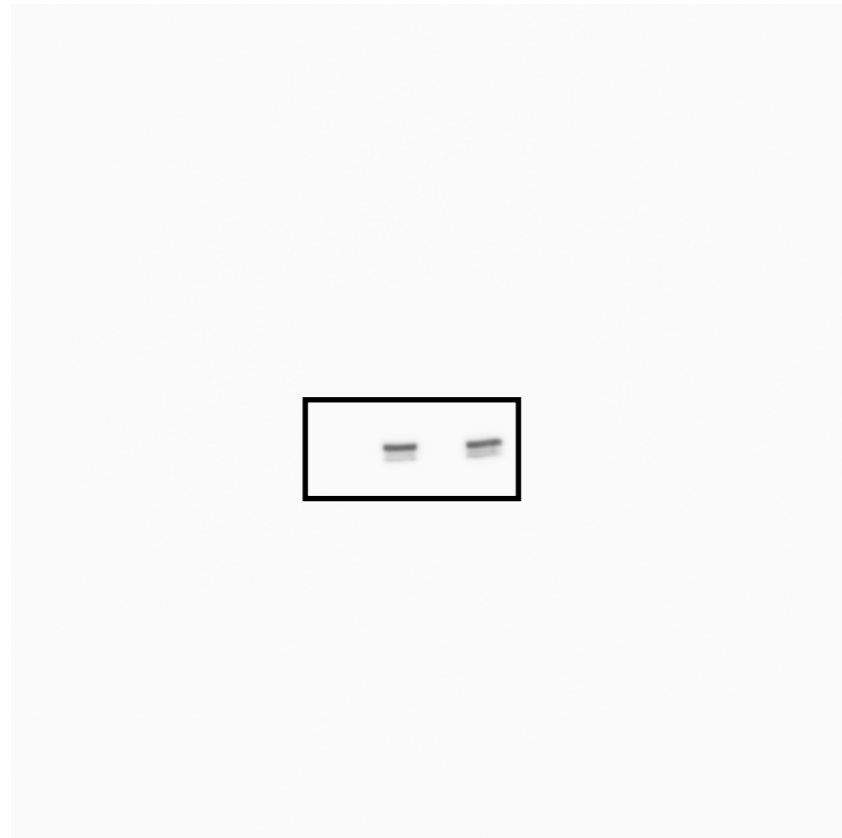

Ago2

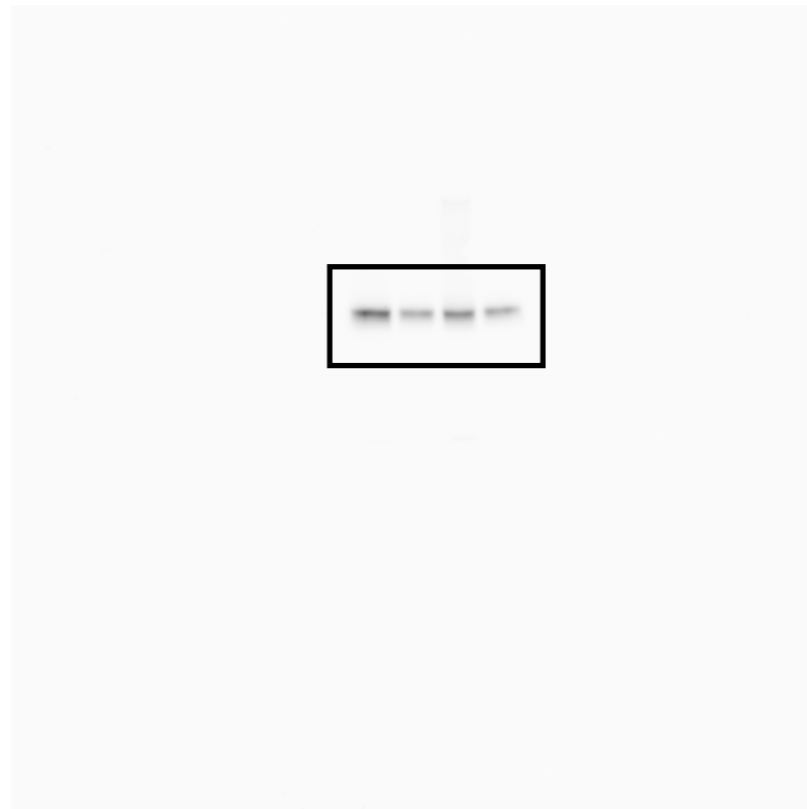

Gapdh

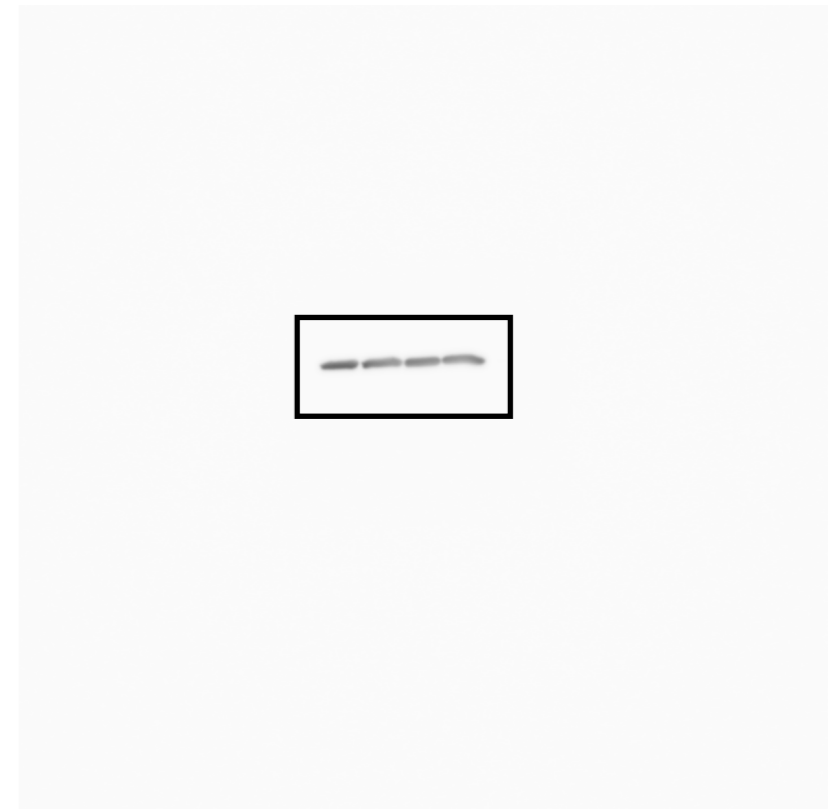

Supplement: Figure 5—source data 1. — An Excel file of numbers for Figure 5C and D. [file elife-72289-fig5-data1.zip › Figure5/Uncropped_Figures/Figure5A_uncropped.pdf]
